# On the eigenvalue effective size of structured populations

**DOI:** 10.1007/s00285-014-0832-5

**Published:** 2014-09-18

**Authors:** Ola Hössjer

**Affiliations:** Divsion of Mathematical Statistics, Department of Mathematics, Stockholm University, Stockholm, Sweden

**Keywords:** Eigenvalue effective size, Coalescence theory, Predicted gene diversity, Migration, Perron–Frobenius, Perturbation theory of eigenvalues, 92D25, 60F99

## Abstract

A general theory is developed for the eigenvalue effective size ($N_{eE}$) of structured populations in which a gene with two alleles segregates in discrete time. Generalizing results of Ewens (Theor Popul Biol 21:373–378, 1982), we characterize $N_{eE}$ in terms of the largest non-unit eigenvalue of the transition matrix of a Markov chain of allele frequencies. We use Perron–Frobenius Theorem to prove that the same eigenvalue appears in a linear recursion of predicted gene diversities between all pairs of subpopulations. Coalescence theory is employed in order to characterize this recursion, so that explicit novel expressions for $N_{eE}$ can be derived. We then study $N_{eE}$ asymptotically, when either the inverse size and/or the overall migration rate between subpopulations tend to zero. It is demonstrated that several previously known results can be deduced as special cases. In particular when the coalescence effective size $N_{eC}$ exists, it is an asymptotic version of $N_{eE}$ in the limit of large populations.

## Introduction

The effective size $N_e$ was introduced by Wright (1931, 1938) as the size of an ideal homogeneous population with the same rate of loss of heterozygosity per generation as the studied population. It has become one of the most important parameters in population genetics and conservation biology, as reviewed for instance by Crow and Denniston (1988), Orrive (1993), Caballero (1994), Wang and Caballero (1999), Waples (2002) and Charlesworth (2009).

Several closely related variants of $N_e$ exist, and Crow (1954) first distinguished between the inbreeding effective size $N_{eI}$, the quantity originally defined by Wright, and the variance effective size $N_{eV}$. He also introduced a random extinction parameter that quantifies the long term rate at which genetic variants are lost. It is equivalent to the eigenvalue effective size $N_{eE}$, defined in terms of the largest non-unit eigenvalue of a Markov chain of allele frequencies (Ewens 1982, 2004). The nucleotide diversity or mutation effective size $N_{e\pi }$ is essentially the expected coalescence time of a pair of haploid individuals (Ewens 1989; Durrett 2008), whereas the coalescent effective size $N_{eC}$ is defined for populations such that the ancestral tree of any finite number of individuals converges to a Kingman coalescent in the limit of large populations (Kingman 1982; Nordborg and Krone 2002; Sjödin et al. 2005; Wakeley and Sargsyan 2009; Hössjer 2011).

In this paper we provide a general theory of $N_{eE}$ for mutation free structured populations, in which a selectively neutral marker (referred to as a gene) with two variants or alleles segregates. The population consists of $s$ homogeneous subpopulations (geographic sites, age classes, sexes or combinations thereof) and evolves in discrete time, with constant census sizes of all subpopulations.

Ewens (2004) reviewed results on $N_{eE}$ for homogeneous populations, showing that it agrees with $N_{eI}$ and $N_{eV}$ for the Wright-Fisher model (Wright 1931; Fisher 1958), Kimura’s multi-hypergeometric model (Kimura 1957), conditional branching process models (Karlin and McGregor 1965) or more generally models in which offspring numbers are exchangeable (Cannings 1974).

Results on $N_{eE}$ for structured populations are less complete. Ewens (1982) showed that $N_{eE}$ may differ from $N_{eI}$ and $N_{eV}$ for two-sex models. Cabellero and Hill (1992) and Nagylaki (1995) considered a number of diploid models and derived formulas for an effective size based on the long term decay of heterozygosity. Chesser et al. (1993) and Wang (1997a, b) analyzed the island model with two sexes. They derived linear recursion formulas for the inbreeding coefficient and the coancestry of individuals from the same and different subpopulations, and computed an effective size from the largest eigenvalue of this recursion. Felsenstein (1971) computed the effective size for models with $s$ age classes, and found the effective size from the largest eigenvalue of a linear recursion of $s^2$ non-identity by descent probabilities of genes drawn with replacement from all pairs of age groups. Maruyama (1970a) derived a similar effective number for the circular stepping stone model under large population and small migration rate limits. Tufto et al. (1996) and Tufto and Hindar (2003) defined the eigenvalue effective size from a linear recursion of covariances between of all pairs of subpopulations.

All these notions of effective size are derived in terms the largest eigenvalue $\lambda$ of linear recursions of covariances or probabilities of identity by descent or state. Whitlock and Barton (1997) showed that these linear recursions are closely related. They also argued briefly that the transition matrix of the Markov chain of allele frequencies has its largest non-unit eigenvalue equal to $\lambda$, and therefore all effective sizes of the previous paragraph agree with $N_{eE}$.

Motivated by the argument of Whitlock and Barton (1997), our main purpose in this paper is to provide a general framework for exact and asymptotic computation of $N_{eE}$ for a large class of structured populations with stochastic backward migration and exchangeable reproduction within subpopulations. In Sect. 2 we introduce the population genetic model and Ewens’ definition of $N_{eE}$ in terms of the rate at which the Markov process of allele frequencies in all subpopulations reaches an absorbing state, quantified by the largest non-unit eigenvalue $\lambda$ of its transition matrix. In Sect. 3 we focus on gene diversities, i.e. probabilities of genes not being identical by state. We introduce an $s^2$-dimensional deterministic process of predicted gene diversities and prove that whenever it is a linear recursion, $\lambda$ also equals the largest eigenvalue of the matrix ${\varvec{A}}$ of this recursion. In Sect. 4 we show how the elements of ${\varvec{A}}$ are obtained from coalescence theory in new settings that generalize previous work, as illustrated with several examples in Sect. 5. Asymptotic approximations of $\lambda$ and $N_{eE}$ are obtained from perturbation theory of eigenvalues of matrices in Sect. 6, when either the population gets large and/or the migration rate gets small. This gives novel asymptotic expressions for $N_{eE}$ that, for instance, in the limit of large populations agrees with the coalescence effective size $N_{eC}$ when the latter exists. A discussion follows in Sect. 7 and proofs are collected in the “Appendix”.

## Model of reproduction, migration and allele frequency change

Consider a population of $N$ individuals, divided into $s$ subpopulations

1 $$\begin{aligned} \mathcal{I}= \{1,\ldots ,s\} \end{aligned}$$

of constant sizes $N_1=Nu_1,\ldots ,N_s=Nu_s$, with $u_i\ge 0$ and $\sum _{i\in \mathcal{I}} u_i = 1$. Each individual carries two copies of a selectively neutral gene so that subpopulation $i$ has a total of $2N_i$ genes.

The population evolves in discrete time (not necessarily generations) $t=0,1,\ldots$, with the genes of each subpopulation $k\in \mathcal{I}$ at time $t-1$ numbered $g=1,\ldots ,2N_k$, and $\nu _{tkig}$ referring to the number of offspring of gene $g$ that migrate to subpopulation $i$ at time $t$. The total gene flow from $k$ to $i$ between $t-1$ and $t$ is summarized by the backward migration rate

2 $$\begin{aligned} \mathcal{B}_{tik} = \frac{1}{2N_i}\sum _{g=1}^{2N_k} \nu _{tkig}, \end{aligned}$$

i.e. the fraction of genes a time $t$ and subpopulation $i$ that originate from $k$ at time $t-1$. The matrix ${\varvec{\mathcal {B}}}_t=(\mathcal{B}_{tij})$ is referred to as the *observed* backward migration matrix at time $t$. Since its row sums are one, it is the transition matrix of a Markov chain with state space $\mathcal{I}$.

Let ${\varvec{\nu }}_{tkg}=(\nu _{tk1g},\ldots ,\nu _{tksg})$ summarize the frequency distribution of the offspring of gene $g$ of subpopulation $k$ at time $t-1$ in all subpopulations. Assume that $\{{\varvec{\nu }}_{tkg}\}_{g=1}^{2N_k}$ are exchangeable random vectors, conditionally on $\varvec{\mathcal {B}}_t$, and that $\{\varvec{\mathcal {B}}_t\}$ are independent and identically distributed random matrices with

3 $$\begin{aligned} E(\varvec{\mathcal {B}}_t) = {\varvec{B}}, \end{aligned}$$

where ${\varvec{B}}=(B_{ik})$, the expected backward migration matrix, is the transition matrix of a Markov chain with state space $\mathcal{I}$. We assume that the ${\varvec{B}}$ is irreducible and aperiodic, with a unique equilibrium distribution ${\varvec{\gamma }}= (\gamma _1,\ldots ,\gamma _s)$ satisfying $\gamma _i\ge 0$, $\sum _{i=1}^s \gamma _i = 1$ and

4 $$\begin{aligned} {\varvec{\gamma }}= {\varvec{\gamma }}{\varvec{B}}. \end{aligned}$$

It follows from (2) that the observed forward migration rate between $k$ and $i$, i.e. the expected number of offspring of the genes in subpopulation $k$ at time $t-1$ that at time $t$ end up in subpopulation $i$, is

5 $$\begin{aligned} \mathcal{M}_{tki} := E(\nu _{tkig}|\varvec{\mathcal {B}}_t) = \frac{u_i\mathcal{B}_{tik}}{u_k}. \end{aligned}$$

In order to keep subpopulation sizes constant over time, the total contribution in (5) from all parental populations $k$ must be constant, i.e.

6 $$\begin{aligned} u_i = \sum _{k=1}^s u_k \mathcal{M}_{tki}. \end{aligned}$$

Let ${\varvec{\mathcal {M}}}_t = (\mathcal{M}_{tki})$ be the observed forward migration matrix of time $t$. It follows from (3) and (5) that the corresponding expected forward migration matrix ${\varvec{M}}=E(\varvec{\mathcal {M}}_t)$ has elements $M_{ki}=E(\nu _{tkig})$ related to those of ${\varvec{B}}$ as

7 $$\begin{aligned} B_{ik} = \frac{u_k M_{ki}}{u_i} \end{aligned}$$

for $1\le k,i \le s$. Taking expectations on both sides of (6), we find that the vector ${\varvec{u}}=(u_1,\ldots ,u_s)$ of relative subpopulation proportions satisfies

8 $$\begin{aligned} {\varvec{u}}= {\varvec{u}}{\varvec{M}}; \end{aligned}$$

a left eigenvector of ${\varvec{M}}$ with eigenvalue 1. The two vectors ${\varvec{u}}$ and ${\varvec{\gamma }}$ are identical for conservative migration (Nagylaki 1980), but in general they differ.

Consider a biallelic genetic marker, and let ${\varvec{X}}_t = (X_{t1},\ldots ,X_{ts})^\prime$ be a column vector of (relative) frequencies of one of the two alleles in all subpopulations at time $t$, where prime denotes transposition. Since $\{{\varvec{\nu }}_{tkg}\}_{g=1}^{2N_k}$ are exchangeable, we may number the genes of subpopulation $k$ and time $t-1$ so that the first $2N_kX_{t-1,k}$ have the specified allele. Then the allele frequency drift from one time point to the next can be summarized as

9 $$\begin{aligned} X_{ti} = \frac{1}{2N_i}\sum _{k=1}^s \sum _{g=1}^{2N_kX_{t-1,k}} \nu _{tkig}. \end{aligned}$$

The following result is a simple consequence of (3), (5) and (9):

### **Proposition 1**

Assume that $\mathcal{B}_t$ is independent of $\{{\varvec{X}}_{s}\}_{s\le t-1}$. Then the sequence $\{{\varvec{X}}_t\}$ of allele frequencies satisfies

10 $$\begin{aligned} E({\varvec{X}}_{t}|{\varvec{X}}_{t-1}={\varvec{x}}) = {\varvec{B}}{\varvec{x}}, \end{aligned}$$

where ${\varvec{x}}$ is a column vector of allele frequencies of length $s$.

We can rephrase Proposition 1 as $\{{\varvec{X}}_t\}$ being a vector-valued autoregressive process of order 1 (Brockwell and Davis 1991). This process will be heteroscedastic, since the covariance matrix $\text{ Var }({\varvec{X}}_{t}|{\varvec{X}}_{t-1}={\varvec{x}})$ varies with ${\varvec{x}}$. The dynamics of $\{{\varvec{X}}_t;\, t\ge 0\}$ is described more generally by means of a time homogeneous Markov chain with a state space

$$\begin{aligned} \mathcal{X}= \left\{ 0,\frac{1}{2N_1},\frac{2}{2N_1},\ldots ,1\right\} \times \cdots \times \left\{ 0,\frac{1}{2N_s},\frac{2}{2N_s},\ldots ,1\right\} \end{aligned}$$

of size $|\mathcal{X}|= \prod _{i=1}^s (2N_i +1)$, and a transition kernel ${\varvec{P}}= \left( P({\varvec{x}},{\varvec{y}})\right)$, with elements $P({\varvec{x}},{\varvec{y}}) = P({\varvec{X}}_{t}={\varvec{y}}|{\varvec{X}}_{t-1}={\varvec{x}})$ for all ${\varvec{x}},{\varvec{y}}\in \mathcal{X}$. Since our model is free of mutations and no subpopulation is isolated, sooner or later one of the two alleles will be fixed in all subpopulations. This can be phrased as $\{{\varvec{X}}_t\}$ having two absorbing states ${\varvec{0}}=(0,\ldots ,0)$ and ${\varvec{1}}=(1,\ldots ,1)$, so that ${\varvec{P}}$ is reducible with two stationary distributions $\pi _1({\varvec{x}}) = 1_{\{{\varvec{0}}\}}({\varvec{x}})$ and $\pi _2({\varvec{x}}) = 1_{\{{\varvec{1}}\}}({\varvec{x}})$, one for each of the absorbing states, with $1_{\mathcal{Y}}({\varvec{x}})$ the indicator function of $\mathcal{Y}\subset \mathcal{X}$. Write ${\varvec{\pi }}_i = (\pi _i({\varvec{x}});\, {\varvec{x}}\in \mathcal{X})$ for the corresponding two row vectors of length $|\mathcal{X}|$. Since ${\varvec{\pi }}_i = {\varvec{\pi }}_i{\varvec{P}}$ for $i=1,2$, they are left eigenvectors of ${\varvec{P}}$ with eigenvalue 1. We divide $\mathcal{X}= \cup _{i=1}^n \mathcal{X}_i$ into components $\mathcal{X}_1=\{{\varvec{0}}\},\mathcal{X}_2=\{{\varvec{1}}\},\mathcal{X}_3,\ldots ,\mathcal{X}_n$ that induce a block form

11 $$\begin{aligned} {\varvec{P}}= \left( \begin{array}{c@{\quad }c@{\quad }c@{\quad }c@{\quad }c} 1 &{} 0 &{} {\varvec{0}}&{} \ldots &{} {\varvec{0}}\\ 0 &{} 1 &{} {\varvec{0}}&{} \ldots &{} {\varvec{0}}\\ {\varvec{P}}_{31} &{} {\varvec{P}}_{32} &{} {\varvec{P}}_{33} &{} \ldots &{} {\varvec{0}}\\ \vdots &{} &{} &{} \ddots &{} \vdots \\ {\varvec{P}}_{n1} &{} {\varvec{P}}_{n2} &{} {\varvec{P}}_{n3} &{} \ldots &{} {\varvec{P}}_{nn} \end{array}\right) \end{aligned}$$

of the transition matrix, with zero blocks above the diagonal. For any function $\phi : \mathcal{X}\rightarrow {\mathbb {R}}$, let $\phi = (\phi ({\varvec{x}});{\varvec{x}}\in \mathcal{X})^\prime$ be a column vector of function values. Then ${\varvec{P}}$ acts as an operator $\phi \rightarrow {\varvec{P}}\phi$ on ${\mathbb {R}}^\mathcal{X}$, as

$$\begin{aligned} (P\phi )({\varvec{x}}) = \sum _{{\varvec{y}}\in \mathcal{X}} P({\varvec{x}},{\varvec{y}})\phi ({\varvec{y}}) = E_{{\varvec{x}}}\left( \phi ({\varvec{X}}_1)\right) , \end{aligned}$$

where $E_{{\varvec{x}}}$ denotes expectation conditionally on ${\varvec{X}}_0={\varvec{x}}$, cf. e.g. Norris (2008). In particular, the column vectors generated from

12 $$\begin{aligned} \phi _1({\varvec{x}})&= 1 - {\varvec{\gamma }}{\varvec{x}},\nonumber \\ \phi _2({\varvec{x}})&= {\varvec{\gamma }}{\varvec{x}}\end{aligned}$$

are both right eigenvectors of ${\varvec{P}}$ with eigenvalue 1, i.e. $\phi _i = {\varvec{P}}\phi _i$ for $i=1,2$. Indeed, this follows from (4) and (10), since

$$\begin{aligned} (P\phi _2)({\varvec{x}}) = E_{{\varvec{x}}} (\gamma {\varvec{X}}_1) = \gamma E_{{\varvec{x}}} ({\varvec{X}}_1) = \gamma {\varvec{B}}{\varvec{x}}= \gamma {\varvec{x}}= \phi _2({\varvec{x}}), \end{aligned}$$

and similarly for ${\varvec{\phi }}_1$.

In order to find the rate of fixation of one of the two alleles, we need to look at ${\varvec{P}}^t$ for large $t$. We apply Markov chain theory and find this rate among all possibly complex-valued eigenvalue of ${\varvec{P}}$, as the largest non-unit one. More specifically, in the “Appendix” we use Perron–Frobenius Theorem (see for instance Cox and Miller 1965) as a main ingredient for establishing the following:

### **Theorem 1**

Suppose the square submatrices ${\varvec{P}}_{ii}$ in (11) along the diagonal are irreducible and aperiodic with at least one row sum less than one, for $i=3,\ldots ,n$. Then the eigenvalues $\lambda _i=\lambda _i({\varvec{P}})$ of ${\varvec{P}}$ (including multiplicity), can be ordered as

13 $$\begin{aligned} 1=\lambda _1=\lambda _2 > \lambda _3 \ge |\lambda _4| \ge \cdots \ge |\lambda _{|\mathcal{X}|}| \ge 0, \end{aligned}$$

with

14 $$\begin{aligned} \lambda _3 = \max _{3\le i \le n} \lambda _{ \text{ max }}({\varvec{P}}_{ii}). \end{aligned}$$

Moreover, if the maximum in (14) is attained uniquely for $i=k$, then

15 $$\begin{aligned} {\varvec{P}}^t = \phi _1\pi _1 + \phi _2\pi _2 + \lambda _3^t \left( \begin{array}{c@{\quad }c@{\quad }c@{\quad }c@{\quad }c} 0 &{} 0 &{} {\varvec{0}}&{} \ldots &{} {\varvec{0}}\\ 0 &{} 0 &{} {\varvec{0}}&{} \ldots &{} {\varvec{0}}\\ {\varvec{R}}_{31} &{} {\varvec{R}}_{32} &{} {\varvec{R}}_{33} &{} \ldots &{} {\varvec{0}}\\ \vdots &{} \vdots &{} \vdots &{} \ddots &{} \vdots \\ {\varvec{R}}_{n1} &{} {\varvec{R}}_{n2} &{} {\varvec{R}}_{n3} &{} \ldots &{} {\varvec{R}}_{nn} \end{array}\right) + o(\lambda _3^t) \end{aligned}$$

as $t\rightarrow \infty$, where ${\varvec{R}}_{ii}={\varvec{0}}$ for $i\ne k$, ${\varvec{R}}_{kk} = \phi _k{\varvec{q}}_k$, ${\varvec{q}}_k=\left( q_k({\varvec{x}});\, {\varvec{x}}\in \mathcal{X}_{k}\right)$ is a row vector and $\phi _k = \left( \phi _k({\varvec{x}}); \,{\varvec{x}}\in \mathcal{X}_k\right) ^\prime$ a column vector, both with strictly positive elements, ${\varvec{R}}_{ij}$ has non-negative elements for $j\ge 3$, and the remainder term is a matrix with all its $|\mathcal{X}|^2$ elements of smaller order than $\lambda _3^t$.

The requirement on ${\varvec{P}}$ in Theorem 1 is very weak, essentially that $\mathcal{X}_3,\ldots ,\mathcal{X}_n$ contain transient states, so that no subpopulation is isolated and eventually one of the two alleles will be fixed in all subpopulations. When $m=3$ there is only one component of transient states, and migration is then possible within a finite number of time steps, back *and* forth between any pair of subpopulations. A recursive formula is provided in the “Appendix” for all ${\varvec{R}}_{ij}$, and for ${\varvec{R}}_{kk}$ we can normalize the two vectors ${\varvec{\phi }}_k$ and ${\varvec{q}}_k$ so that $\sum _{{\varvec{x}}\in \mathcal{X}_k} q_k({\varvec{x}}) = \sum _{{\varvec{x}}\in \mathcal{X}_k} \phi _k({\varvec{x}})q_k({\varvec{x}})=1$. Then ${\varvec{q}}_k$ is the quasi equilibrium distribution

16 $$\begin{aligned} q_k({\varvec{x}}) = \lim _{t\rightarrow \infty } P_\pi ({\varvec{X}}_t={\varvec{x}}|{\varvec{X}}_{t^\prime }\in \mathcal{X}_k,\quad t^\prime = 0,1,\ldots ,t-1) > 0 \end{aligned}$$

of ${\varvec{X}}_t$ conditionally on starting and remaining in $\mathcal{X}_k$ (Darroch and Seneta 1965; Collet and Martinez 2013). The quasi equilibrium distributions for the other $\mathcal{X}_3,\ldots ,\mathcal{X}_n$ are part of the remainder term of (15).

The following important corollary of Theorem 1 deals with the asymptotic decay rate of the expected value of $\phi ({\varvec{X}}_t)$ for a large class of functions:

### **Corollary 1**

Let $\phi :\mathcal{X}\rightarrow {\mathbb {R}}$ be a function satisfying

17 $$\begin{aligned} \begin{array}{rcll} \phi ({\varvec{x}}) &{}=&{} 0, &{} {\varvec{x}}\in \mathcal{X}_1\cup \mathcal{X}_2\\ \phi ({\varvec{x}}) &{}\ge &{} 0, &{} {\varvec{x}}\in \mathcal{X}\setminus (\mathcal{X}_1\cup \mathcal{X}_2\cup \mathcal{X}_k),\\ \phi ({\varvec{x}}) &{} > &{} 0, &{} {\varvec{x}}\in \mathcal{X}_k, \end{array} \end{aligned}$$

where $\mathcal{X}_k$ is the component for which the maximum in (14) is attained. Then

18 $$\begin{aligned} \lim _{t\rightarrow \infty } \frac{E_\pi (\phi ({\varvec{X}}_t))}{\lambda _3^t}&= \sum _{{\varvec{x}},{\varvec{y}}\in \mathcal{X}} \pi ({\varvec{x}}){\varvec{R}}({\varvec{x}},{\varvec{y}})\phi ({\varvec{y}})\nonumber \\&\ge \sum _{{\varvec{x}}\in \mathcal{X}_k} \pi ({\varvec{x}})\phi _k({\varvec{x}}) \cdot \sum _{{\varvec{y}}\in \mathcal{X}_k} q_k({\varvec{y}})\phi ({\varvec{y}}), \end{aligned}$$

where ${\varvec{R}}=({\varvec{R}}_{ij})=\left( R({\varvec{x}},{\varvec{y}})\right)$ is the matrix in (15), and $E_\pi$ denotes expected value conditional on $P_\pi ({\varvec{X}}_0={\varvec{x}})=\pi ({\varvec{x}})$. In particular, the right hand side of (18) is strictly positive if $\pi (\mathcal{X}_k)>0$.

Corollary 1 shows that the largest non-unit eigenvalue $\lambda _3=\lambda _3({\varvec{P}})$ determines the rate of decrease of the expected value $E_\pi (\phi ({\varvec{X}}_t))$ as $t\rightarrow \infty$. Putting $\phi ({\varvec{x}})=1_{\{{\varvec{x}}\notin \mathcal{X}_1\cup \mathcal{X}_2\}}$, we notice that the probability of non-fixation decreases with $t$ at this rate, so that $\lambda _3$ is the rate of fixation and the eigenvalue of main interest. We will often simplify notation and write

19 $$\begin{aligned} \lambda = \lambda _3({\varvec{P}}). \end{aligned}$$

The Wright-Fisher (WF) model is a homogeneous population ($s=1$) of $N$ diploid individuals, with $X_{t}|X_{t-1} \sim \text{ Bin }(2N,X_{t-1})/(2N)$. Feller (1951) found all the eigenvalues of the transition matrix for the WF model, and in particular

20 $$\begin{aligned} \lambda _3({\varvec{P}}_{\mathrm{WF}}) = 1 - \frac{1}{2N}. \end{aligned}$$

For an allele frequency process $\{{\varvec{X}}_t\}$ with transition matrix ${\varvec{P}}$, we define the eigenvalue effective size

21 $$\begin{aligned} N_{eE} = \frac{1}{2(1-\lambda )} = \frac{1}{2(1-\lambda _3({\varvec{P}}))} \end{aligned}$$

as the size of a WF population for which the largest non-unit eigenvalue in (20) is the same as for the studied population.

## Rate of decay of predicted gene diversities

Following Nei (1973, 1987), we define the gene diversity

22 $$\begin{aligned} \mathcal{H}_{tij} = X_{ti}(1-X_{tj}) + X_{tj}(1-X_{ti}) \end{aligned}$$

between subpopulations $i$ and $j$ at time $t$ as the probability that two randomly chosen genes from $i$ and $j$ (picked with replacement if $i=j$) are different by state, i.e. have different types of alleles. Regarding $t=0$ as present and $t>0$ as future, let

23 $$\begin{aligned} H_{tij} = E_\pi (\mathcal{H}_{tij}) \end{aligned}$$

be the predicted gene diversity between $i$ and $j$ at time $t$, given an initial distribution ${\varvec{X}}_0\sim \pi$. We collect the predicted gene diversities between all pairs of subpopulations into a column vector

24 $$\begin{aligned} {\varvec{H}}_t = {\varvec{H}}_t(\pi ) = \text{ vec }\left( (H_{tij})_{i,j=1}^s\right) \end{aligned}$$

of length $s^2$, where vec is the vectorization operator that converts a matrix into a column vector by stacking its columns on top of each other. In order to compute linear combinations of the elements of ${\varvec{H}}_t$, we define weights $W_{ij}$ for all pairs of subpopulations, and prove the following:

### **Proposition 2**

Suppose ${\varvec{W}}= \text{ vec }\left( (W_{ij})_{i,j=1}^s\right) ^\prime$ is a row vector of length $s^2$ with non-negative weights $W_{ij}\ge 0$ satisfying the symmetry condition $W_{ij}=W_{ji}$ for all $i,j$, and let $\phi _{{\varvec{W}}}({\varvec{x}}) = 2\sum _{i,j=1}^s W_{ij}x_i(1-x_j)$ be a quadratic functional of ${\varvec{x}}=(x_1,\ldots ,x_s)^\prime$. Then

$$\begin{aligned} {\varvec{W}}{\varvec{H}}_t = \sum _{i,j=1}^s W_{ij}H_{tij} = E_\pi \left( \phi _{{\varvec{W}}}({\varvec{X}}_t)\right) . \end{aligned}$$

To see the importance of Proposition 2, we notice that a sufficient condition for $\phi _{{\varvec{W}}}$ to satisfy (17) is that all $W_{ij}>0$. It therefore follows from Corollary 1 that we can find $\lambda =\lambda _3({\varvec{P}})$ and hence also $N_{eE}$ from the rate of decrease to $0$ of linear combinations of $H_{tij}$. It is therefore of interest to study the time dynamics of ${\varvec{H}}_t$, and we will assume that a non-negative square matrix ${\varvec{A}}$ of order $s^2$ exists, so that ${\varvec{H}}_t$ satisfies the linear recursion

25 $$\begin{aligned} {\varvec{H}}_{t} = {\varvec{A}}{\varvec{H}}_{t-1} \end{aligned}$$

for $t=1,2,\ldots$. It will be convenient to introduce

26 $$\begin{aligned} \mathcal{I}_2 = \mathcal{I}\times \mathcal{I}, \end{aligned}$$

the set of all pairs of subpopulations (cf. (1)), and write the elements of ${\varvec{A}}$ as

27 $$\begin{aligned} {\varvec{A}}=(A_{ij,kl})_{ij\in \mathcal{I}_2, kl\in \mathcal{I}_2}, \end{aligned}$$

where $ij$ and $kl$ is short hand notation for the row and column numbers obtained from the stacking procedure of the vec operation. We can always divide (26) into $m$ irreducible components $\mathcal{I}_2 = \mathcal{C}_1\cup \cdots \cup \mathcal{C}_m$. After a possible reordering of the elements of $\mathcal{I}_2$, this gives a corresponding block decomposition

28 $$\begin{aligned} {\varvec{A}}= \left( \begin{array}{c@{\quad }c@{\quad }c@{\quad }c} {\varvec{A}}_{11} &{} {\varvec{0}}&{} \ldots &{} {\varvec{0}}\\ {\varvec{A}}_{21} &{} {\varvec{A}}_{22} &{} \ldots &{} {\varvec{0}}\\ \vdots &{} &{} \ddots &{} \vdots \\ {\varvec{A}}_{m1} &{} {\varvec{A}}_{m2} &{} \ldots &{} {\varvec{A}}_{mm} \end{array}\right) \end{aligned}$$

of (27), with ${\varvec{A}}_{aa}=(A_{ij,kl})_{ij,kl\in \mathcal{C}_a}$ irreducible for $a=1,\ldots ,m$. Typically, the pair of ancestors of two genes, picked from any pair $\mathcal{I}_2$ of subpopulations, will ultimately belong to $\mathcal{C}_1$, provided the ancestry is traced sufficiently far back in time. The following fundamental result clarifies the importance of ${\varvec{A}}$:

### **Theorem 2**

Let $\{{\varvec{X}}_t\}$ satisfy the conditions of Theorem 1, with $\lambda =\lambda _3({\varvec{P}})$ the largest non-unit eigenvalue of its transition matrix ${\varvec{P}}$, defined in (19). Assume that (25) holds, with ${\varvec{A}}$ having non-negative elements. Then

29 $$\begin{aligned} \lambda = \lambda _{ \text{ max }}({\varvec{A}}) = \max _{1\le a \le m} \lambda _{ \text{ max }}({\varvec{A}}_{aa}), \end{aligned}$$

where ${\varvec{A}}_{aa}$ are the diagonal matrices of (28). If the maximum in (29) is attained for a unique $1\le c\le m$, then

30 $$\begin{aligned} {\varvec{A}}^t = \lambda ^t {\varvec{r}}{\varvec{\rho }} + o(\lambda ^t) \text{ as } t\rightarrow \infty , \end{aligned}$$

with ${\varvec{\rho }} =\text{ vec }\left( (\rho _{ij})_{ij\in \mathcal{I}_2}\right) ^\prime$ and ${\varvec{r}}= \text{ vec }\left( (r_{ij})_{ij\in \mathcal{I}_2}\right)$ left and right eigenvectors

31 $$\begin{aligned} {\varvec{\rho }}{\varvec{A}}&= \lambda {\varvec{\rho }},\nonumber \\ {\varvec{A}}{\varvec{r}}&= \lambda {\varvec{r}}\end{aligned}$$

of ${\varvec{A}}$ with eigenvalue $\lambda$. Explicit expressions for ${\varvec{\rho }}$ and ${\varvec{r}}$ are provided in the “Appendix”, and their components can be normalized so that $\rho _{ij},r_{ij}>0$ for $ij\in \mathcal{C}_c$, $\sum _{ij\in \mathcal{C}_c} \rho _{ij} = \sum _{ij\in \mathcal{C}_c} \rho _{ij}r_{ij}=1$, $\rho _{ij}=0$ for $ij\in \mathcal{C}_{c+1}\cup \cdots \cup \mathcal{C}_m$ and $r_{ij}=0$ for $ij\in \mathcal{C}_{1}\cup \cdots \cup \mathcal{C}_{c-1}$.

The following key result follows from (21) and Theorems 1–2:

### **Corollary 2**

Suppose $\{{\varvec{X}}_t\}$ satisfies the conditions of Theorem 1, with a linear recursion (25) for predicted gene diversities in terms of a non-negative quadratic matrix ${\varvec{A}}$. Then the eigenvalue effective size is

32 $$\begin{aligned} N_{eE} = \frac{1}{2(1-\lambda _{ \text{ max }}({\varvec{A}}))}. \end{aligned}$$

## Coalescence probabilities

In this section we derive a linear recursion (25) for the predicted gene diversity vector ${\varvec{H}}_t$, using probabilities that ancestral lineages of two genes coalesce. This enables us to compute $N_{eE}$ from (32).

Many authors have derived linear recursions for identity by descent probabilities, gene diversities, covariances or coalescence probabilities of subdivided populations with spatial, age, sex or some other structure, with or without mutations. This includes results in Malécot (1951), Hill (1972), Li (1976), Sawyer (1976), Felsenstein (1982), Slatkin (1991), Nagylaki (2000), Ryman and Leimar (2008), Durrett (2008), Hössjer and Ryman (2014), Hössjer et al. (2014) and other papers mentioned in the introduction. They utilize coalescence probabilities more or less explicitly. We will generalize several of these results for constant subpopulation sizes, allowing backward migration rates to be stochastic and reproduction within subpopulations to have a general form.

Consider a structured coalescent (Notohara 1990; Herbots 1997; Wakeley 1998) for two genes, with coalescence events formulated hierarchically in two steps, first for subpopulations and then for genes. We draw two different genes from the population at time $t$ and consider their joint ancestral subpopulation history $(I_\tau ,J_\tau )_{\tau =0}^T$ at times $\{t-\tau \}_{\tau =0}^T$ until they find their most recent common ancestor at $t-T$, where $T$ is the coalescence time. Let

33 $$\begin{aligned} \mathcal{Q}_{t,ij,kl} = P\left( (I_{1},J_{1})=(k,l)|(I_{0},J_{0})= (i,j),T>0,\varvec{\mathcal {B}}_{t}\right) \end{aligned}$$

be the probability that the parents of two genes drawn from subpopulations $i$ and $j$ at time $t$ have parents from subpopulations $k$ and $l$, conditionally on $\varvec{\mathcal {B}}_{t}$. Since there are $2Nu_i\mathcal{B}_{tik}$ genes of subpopulation $i$ at time that originate from subpopulation $k$, it follows that

34 $$\begin{aligned} \mathcal{Q}_{tij,kl} = \left\{ \begin{array}{l@{\quad }l} \mathcal{B}_{tik}\mathcal{B}_{tjl}, &{} i\ne j,\\ 2Nu_i\mathcal{B}_{tik}(2Nu_i\mathcal{B}_{tik}-1)/(2Nu_i(2Nu_i-1)), &{} i=j, k=l, \\ 2Nu_i\mathcal{B}_{tik} \cdot 2Nu_i\mathcal{B}_{til}/(2Nu_i(2Nu_i-1)), &{} i=j, k\ne l. \end{array}\right. \qquad \end{aligned}$$

We gather all these probabilities into an observed backward migration matrix $\varvec{\mathcal {Q}}_{t} =(\mathcal{Q}_{t,ij,kl})_{ij\in \mathcal{I}_2,kl\in \mathcal{I}_2}$ of order $s^2$ for pairs of subpopulations. By averaging with respect to $\varvec{\mathcal {B}}_{t}$, we then define the unconditional probabilities

35 $$\begin{aligned} Q_{ij,kl} = E(\mathcal{Q}_{t,ij,kl}) = P\left( (I_{1},J_{1})= (k,l)|(I_{0},J_{0})=(i,j),T>0\right) \end{aligned}$$

that the parents of two genes from $i$ and $j$ belong to $k$ and $l$, and collect them into a square matrix ${\varvec{Q}}=(Q_{ij,kl})_{ij\in \mathcal{I}_2,kl\in \mathcal{I}_2}$ of order $s^2$. The following result summarizes the role of $\varvec{\mathcal {Q}}_t$ and ${\varvec{Q}}$ and is stated without proof:

### **Proposition 3**

Suppose segregation is independent between generations and condition on that coalescence events do not take place. Then the joint subpopulation ancestry $\{(I_\tau ,J_\tau )\}_{\tau =0}^t$ of a pair of different genes from time $t$ back to time 0 is a Markov chain with state space $\mathcal{I}_2$ that (a) conditionally on $\{\varvec{\mathcal {B}}_{t-\tau }; \, \tau \ge 0\}$ has time varying transition matrices $\{\varvec{\mathcal {Q}}_{t-\tau }\}_{\tau =0}^{t-1}$, (b) unconditionally has a time invariant transition matrix ${\varvec{Q}}$.

The next step is to incorporate coalescence events. To this end, let

36 $$\begin{aligned} \mathcal{P}_{t,ijk}=P\left( T=1|I_{0}=i,J_{0}=j,I_{1}=J_{1}=k,\varvec{\mathcal {B}}_{t}, T>0\right) \end{aligned}$$

be the probability that two genes from subpopulations $i$ and $j$ at time $t$ have the same parent, given that the parent belongs to $k$, and conditionally on $\varvec{\mathcal {B}}_{t}$. The corresponding unconditional coalescence probability $p_{ijk}$ that two genes from $i$ and $j$ that both have their parents in $k$, have the same parent, is

37 $$\begin{aligned} p_{ijk} = P(T=1|I_0=i,J_0=j,I_1=J_1=k,T>0). \end{aligned}$$

It will be helpful to introduce the quantities

38 $$\begin{aligned} V_{kij} = \left\{ \begin{array}{ll} E(\nu _{tki1}(\nu _{tki1}-1)), &{} \text{ if } \,i=j,\\ E(\nu _{tki1}\nu _{tkj1}), &{} \text{ if } \,i\ne j, \end{array}\right. \end{aligned}$$

in order to get a more explicit expression for $p_{ijk}$, where $\nu _{tkig}$ in (2) is the number of offspring of gene $g$ in subpopulation $k$ at time $t-1$ that end up in $i$ at time $t$. The variables $\{V_{kij}\}_{i,j=1}^s$ quantify the average variability of reproductive success among individuals in subpopulation $k$, and the coalescence probabilities are closely related to standardized versions of them:

### **Theorem 3**

Suppose the backward subpopulation ancestry of two different genes before coalescence is a Markov chain, with transition matrix ${\varvec{Q}}=(Q_{ij,kl})$ as in (35), and define coalescence probabilities $p_{ijk}$ as in (37). Then

39 $$\begin{aligned} p_{ijk} = \left( \frac{1}{1-\frac{1}{2Nu_i}}\right) ^{\{i=j\}} \frac{V_{kij}u_k}{2Nu_iu_jQ_{ij,kk}}, \end{aligned}$$

with $V_{kij}$ defined in (38), and the components (22) of the gene diversity vectors ${\varvec{\mathcal {H}}}_t = \text{ vec }\left( (\mathcal{H}_{tij})_{i,j=1}^s\right)$ form a multivariate autoregressive process

40 $$\begin{aligned} \mathcal{H}_{tij} = \sum _{k,l=1}^s A_{ij,kl}\mathcal{H}_{t-1,kl} + \epsilon _{tij} \end{aligned}$$

of order one for $t=0,1,\ldots$, with $\epsilon _{tij}$ satisfying $E(\epsilon _{tij}|{\varvec{X}}_{t-1})=0$, and

41 $$\begin{aligned} A_{ij,kl} = \left( 1-\frac{1}{2Nu_i}\right) ^{\{i=j\}} \left( \frac{1-p_{ijk}}{1-\frac{1}{2Nu_k}}\right) ^{\{k=l\}} Q_{ij,kl}. \end{aligned}$$

The expected gene diversity ${\varvec{H}}_t$ satisfies (25), with ${\varvec{A}}=(A_{ij,kl})$ as in (41).

It turns out that the right eigenvector of ${\varvec{P}}$ corresponding to its third largest eigenvalue $\lambda _3$ can be found explicitly, whenever (40) holds:

### **Corollary 3**

Suppose the conditions of Theorems 1 and 2 hold, with a gene diversity process $\varvec{\mathcal {H}}_t$ satisfying (40). Then the transition matrix ${\varvec{P}}$ of the Markov chain ${\varvec{X}}_t$ has a right eigenvector $\phi _3=(\phi _3({\varvec{x}});\, {\varvec{x}}\in \mathcal{X})^\prime$, corresponding to the third largest eigenvalue $\lambda =\lambda _3({\varvec{P}})$, with

42 $$\begin{aligned} \phi _3({\varvec{x}}) = \sum _{i,j=1}^s\rho _{ij}\left( x_i(1-x_j)+x_j (1-x_i)\right) , \end{aligned}$$

where ${\varvec{\rho }} = (\rho _{ij})$ is the left eigenvector (14) corresponding to the largest eigenvalue $\lambda$ of ${\varvec{A}}=(A_{ij,kl})$. Moreover,

43 $$\begin{aligned} \phi _3({\varvec{x}}) = \left\{ \begin{array}{l@{\quad }l} 0, &{} {\varvec{x}}\in \mathcal{X}_1 \cup \cdots \cup \mathcal{X}_{k-1},\\ >0, &{} {\varvec{x}}\in \mathcal{X}_k,\\ \ge 0, &{} {\varvec{x}}\in \mathcal{X}_{k+1}\cup \cdots \cup \mathcal{X}_n, \end{array}\right. , \end{aligned}$$

where $\mathcal{X}_k$ is the component of $\mathcal{X}_1\cup \cdots \mathcal{X}_n$ for which the maximum in (14) is uniquely attained. In particular, the restriction of $\phi _3$ to $\mathcal{X}_k$ agrees (up to a multiplicative constant) with the function $\phi _k$ of Theorem 1.

## Examples

The key formula (41) provides a general way to find ${\varvec{A}}$ and hence also $N_{eE}$ through (32). We study its two main ingredients; expected backward migration rates $Q_{ij,kl}$ for pairs of genes in Subsect. 5.1, and coalescence probabilities $p_{ijk}$ in Subsect. 5.2. Then we apply these findings to a number of models in Subsect. 5.3 in order to show the generality of Theorem 3, explain how to apply it and as a preparation for the asymptotics of Sect. 6.

### Backward migration

#### *Example 1*

*(Fixed backward migration)*. When the observed backward migration rates are non-random, we must have

44 $$\begin{aligned} \varvec{\mathcal {B}}_t={\varvec{B}}\end{aligned}$$

in order to satisfy (3). It follows from (34), (35) and (44) that

45 $$\begin{aligned} Q_{ij,kl} = \mathcal{Q}_{tij,kl} = \left\{ \begin{array}{l@{\quad }l} B_{ik}B_{jl}, &{} i\ne j,\\ B_{ik}(2Nu_iB_{ik}-1)/(2Nu_i-1), &{} i=j, k=l, \\ 2Nu_i B_{ik} B_{il}/(2Nu_i-1), &{} i=j, k\ne l. \end{array}\right. \end{aligned}$$

Following the nomenclature of Sved and Latter (Sved and Latter (1977)), we refer to (44) as fixed migration rates. $\square$

#### *Example 2*

*(Dirichlet multinomial backward migration)*. Denote the $i$:th rows of ${\varvec{B}}$ and $\varvec{\mathcal {B}}_t$ by ${\varvec{B}}_i = (B_{i1},\ldots ,B_{is})$ and $\varvec{\mathcal {B}}_{ti}=(\mathcal{B}_{ti1},\ldots ,\mathcal{B}_{tis})$ respectively. Specify parameters $0< \alpha _i< \infty$, a random matrix $\check{\varvec{\mathcal {B}}}_t$ with rows

46 $$\begin{aligned} \check{\varvec{\mathcal {B}}}_{ti} \sim \text{ Dir }(\alpha _i{\varvec{B}}_i) \end{aligned}$$

that are independent for $i=1,\ldots ,s$, and assume

47 $$\begin{aligned} \varvec{\mathcal {B}}_{ti}|\check{\varvec{\mathcal {B}}}_{ti} \sim \frac{\text{ Mult }(2Nu_i, \check{\varvec{\mathcal {B}}}_{ti})}{2Nu_i} \end{aligned}$$

are conditionally independent and multinomially distributed random vectors, given all $\check{\varvec{\mathcal {B}}}_{ti}$. Combining (46) and (47), the rows of $\varvec{\mathcal {B}}_t$ are independent random vectors with Dirichlet multinomial distributions. The parameters $\alpha _i$ quantify the amount of variability of the rows of $\check{\varvec{\mathcal {B}}}_t$. We will also extend (46) to $\alpha _i\in \{0,\infty \}$, which in conjunction with (47) gives two degenerate cases within the Dirichlet multinomial family: When $\alpha _i\equiv \infty$, $\check{\varvec{\mathcal {B}}}_t={\varvec{B}}_t$ is fixed, and

48 $$\begin{aligned} \varvec{\mathcal {B}}_{ti} \sim \frac{\text{ Mult }(2Nu_i,{\varvec{B}}_{i})}{2Nu_i}, \end{aligned}$$

whereas if $\alpha _i\equiv 0$ we have

49 $$\begin{aligned} \varvec{\mathcal {B}}_{ti} = \check{\varvec{\mathcal {B}}}_{ti} \sim \text{ Mult }(1,{\varvec{B}}_i), \end{aligned}$$

so that when time proceeds backwards, the ancestral history of *all* genes within a subpopulation will vary according to the same Markov chain with transition matrix ${\varvec{B}}$. From a forward perspective, the latter system has $s$ subpopulation, but the backwards scenario will be identical to that of a single population whose size varies between $2Nu_1,\ldots ,2Nu_s$, according to a Markov chain with transition kernel ${\varvec{B}}$, see for instance Jagers and Sagitov (2004), Sampson (2006), Pollak (2010), Kaj and Krone (2003) and Sano et al. (2004).

For any $0\le \alpha _1,\ldots ,\alpha _s \le \infty$, we notice that

$$\begin{aligned} E(\varvec{\mathcal {B}}_t) = E\left( E(\varvec{\mathcal {B}}_t|\check{\varvec{\mathcal {B}}}_t)\right) = E(\check{\varvec{\mathcal {B}}}_t) = {\varvec{B}}, \end{aligned}$$

in accordance with (3). Since two genes of subpopulations $i$ and $j$ are drawn independently with multinomial distributions from rows $i$ and $j$ of $\check{\varvec{\mathcal {B}}}_t$, it follows from (34) that $E\left( \mathcal{Q}_{tij,kl}|{\varvec{\check{\mathcal{B}}}}_t\right) = \check{\mathcal{B}}_{tik}\check{\mathcal{B}}_{tjl}$. Since the rows of $\check{\varvec{\mathcal {B}}}_t$ have independent Dirichlet distributions (46);

50 $$\begin{aligned} Q_{ij,kl}&= E\left( E(\mathcal{Q}_{tij,kl}|{\varvec{\check{\mathcal{B}}}}_t)\right) \nonumber \\&= E\left( \check{\mathcal{B}}_{tik}\check{\mathcal{B}}_{tjl}\right) \nonumber \\&= E(\check{\mathcal{B}}_{tik})E(\check{\mathcal{B}}_{tjl}) + \text{ Cov }(\check{\mathcal{B}}_{tik},\check{\mathcal{B}}_{tjl})\nonumber \\&= B_{ik}B_{jl} +1_{\{i=j\}}\frac{1_{\{k=l\}}B_{ik}-B_{ik} B_{jl}}{\alpha _i+1}, \end{aligned}$$

which simplifies to

51 $$\begin{aligned} Q_{ij,kl} = B_{ik}B_{jl} \end{aligned}$$

when $\alpha _i\equiv \infty$ and the rows of $\varvec{\mathcal {B}}_t$ have multinomial distributions. $\square$

### Coalescence probabilities

#### *Example 3*

*(Mixed multinomial reproduction)*. Coalescence probabilities require that a reproduction scheme is specified. A fairly general scheme is defined by dividing the time interval between $t-1$ and $t$ into three steps. In a first fertilization step, a gamete pool of infinite size is created for each parental subpopulation $k$, to which the $2N_k$ genes contribute in fractions $\omega _{tk1},\ldots ,\omega _{tk,2N_k}$ that are exchangeable random variables summing to one. In a second migration migration step, the $s$ gamete pools mix, so that the infinitely sized post-migration gamete pool $i$ is a mixture of pre-migration pools $1,\ldots ,s$ in proportions $\check{\mathcal{B}}_{ti1},\ldots ,\check{\mathcal{B}}_{tis}$. In the final reproduction step, subpopulation $k$ at time $t$ is formed by drawing $2N_k$ genes from post migration gamete pool $k$. Then (47) holds, and regardless of the distribution of $\check{\mathcal{B}}_{tik}$,

52 $$\begin{aligned} (\nu _{tki1},\ldots ,\nu _{tki,2N_k})|\varvec{\mathcal {B}}_t,{\varvec{\omega }}_{tk} \sim \text{ Mult }(2N_i\mathcal{B}_{tik},{\varvec{\omega }}_{tk}) \end{aligned}$$

independently between all pairs of parental and offspring subpopulations $k$ and $i$, where ${\varvec{\omega }}_{tk} = (\omega _{tk1},\ldots ,\omega _{tk,2N_k})$.

When (52) holds, we evaluate the expected value in (38) by conditioning on $\mathcal{B}_{tik}$ and ${\varvec{\omega }}_{tk}$ and then insert into (39). By second moment properties of the multinomial distribution; this yields

53 $$\begin{aligned} p_{ijk}&= 2N_k E(\omega _{tk1}^2)\nonumber \\&= (2N_k)^{-1} + 2N_k \text{ Var }(\omega _{tk1})\nonumber \\&=: (2N_{eI,k})^{-1} \end{aligned}$$

and this can also (more easily) be obtained by a direct argument. The coalescence probability $p_{ijk}=p_k$ in (53) only depends on the parental subpopulation $k$, since ${\varvec{\omega }}_{tk}$ is the same, regardless of the offsprings’ subpopulations $i$ and $j$. In the second step of (53) we used $E(\omega _{tk1})=1/(2N_k)$, since the components of ${\varvec{\omega }}_{tk}$ are exchangeable. We interpret $N_{eI,k}\le N_k$ as a local inbreeding effective size of subpopulation $k$, with equality if and only if ${\varvec{\omega }}_{tk}$ is non-random. $\square$

#### *Example 4*

*(Survival indicators)*. Consider the genes $g=1,\ldots ,N_k$ of subpopulation $k$ at time $t-1$, and assume that transitions $k\rightarrow i$ from time $t-1$ to a specific subpopulation $i$ at time $t$ represent their survival, with $\nu _{tkig}\in \{0,1\}$ an indicator for gene $g$ to be still alive at time $t$. Then

54 $$\begin{aligned} p_{iik} = 0 \end{aligned}$$

follows from (38) to (39). For all other subpopulations $j\ne i$ we let $\nu _{tkjg}$ refer to the number of offspring of $g$. Then $V_{kij} = P(\nu _{tki1}=1) E(\nu _{tkj1}| \nu _{tki1}=1) =: M_{ki}M_{k,j|i}$, where $M_{k,j|i}$ is the expected number of offspring in $j$ for a gene that survives, possibly different from the expected number of offspring $M_{kj}=E(\nu _{tkj1})$ of *any* gene in $k$. Since (34)–(35) imply $Q_{ij,kk}=B_{ik}B_{jk}$, it follows from (7) and (39) that the coalescence probability

55 $$\begin{aligned} p_{ijk} = \frac{1}{2Nu_k} \cdot \frac{M_{k,j|i}}{M_{kj}} \end{aligned}$$

is the inverse of the local census size of $k$ times a correction factor that quantifies how correlated survival is with number of progeny in $j$. $\square$

### Example models

#### *Example 5*

*(Cannings models)*. For a homogeneous population ($s=1$), we may drop subpopulation index and write $\nu _{t11g}=\nu _{tg}$, with $\nu _{t1},\ldots ,\nu _{t,2N}$ exchangeable random variables. Since subpopulation sizes and backward migration probabilities are trivial ($u_1=Q_{11,11}=1$), we only need to specify coalescence probabilities $p=p_{111}$. It follows from (38)–(39) that

$$\begin{aligned} p = \frac{E\left( \nu _{t1}(\nu _{t1}-1)\right) }{2N-1}, \end{aligned}$$

and $A=A_{11,11}=1-p$ due to (41). Insertion into (32) yields

56 $$\begin{aligned} N_{eE} = \frac{1}{2(1-A)} = \frac{N-\frac{1}{2}}{E \left( \nu _{t1}(\nu _{t1}-1)\right) }, \end{aligned}$$

in agreement with Section 3.7 of Ewens (2004). Notice that (56) implies $N_{eE}=N$ for a Wright-Fisher model, since $\nu _{t1}\sim \text{ Bin }(2N,1/(2N))$. $\square$

#### *Example 6*

*(Sink and source populations)*. This population has a source of size $N_1$ and a sink of size $N_2$. It is assumed that on average $N_{ \text{ emig }}$ individuals emigrate from 1 to 2 per generation. The migration scheme is degenerate in the sense that the expected forward and backward migration matrices are both reducible;

$$\begin{aligned} {\varvec{M}}= \left( \begin{array}{c@{\quad }c} 1 &{} N_{ \text{ emig }}/N_1 \\ 0 &{} (N_2-N_{ \text{ emig }})/N_2 \end{array}\right) , \quad {\varvec{B}}= \left( \begin{array}{c@{\quad }c} 1 &{} 0\\ N_{ \text{ emig }}/N_2 &{} (N_2-N_{ \text{ emig }})/N_2 \end{array}\right) , \end{aligned}$$

although the equilibrium distribution ${\varvec{\gamma }}=(1,0)$ of ${\varvec{B}}$ is still unique. We assume multinomial backward migration (51), and reproduction where parents are drawn uniformly and independently from the parental subpopulation, corresponding to $p_{ijk}=1/(2N_k)$ in (53). If subpopulations are ordered as $\mathcal{I}_2=\{11,12,21,22\}$, we find from (41) that

$$\begin{aligned} {\varvec{A}}= \left( \begin{array}{c@{\quad }c@{\quad }c@{\quad }c} 1-\frac{1}{2N_1} &{} 0 &{} 0 &{} 0 \\ B_{21} &{} B_{22} &{} 0 &{} 0 \\ B_{21} &{} 0 &{} B_{22} &{} 0 \\ (1-\frac{1}{2N_2})B_{21}^2 &{} (1-\frac{1}{2N_2})B_{21}B_{22} &{} (1-\frac{1}{2N_2})B_{21}B_{22} &{} (1-\frac{1}{2N_2})B_{22}^2 \end{array}\right) . \end{aligned}$$

It is evident that ${\varvec{A}}$ has a block structure (28), with $\mathcal{C}_1=11$, $\mathcal{C}_2 = 12$, $\mathcal{C}_3 = 21$ and $\mathcal{C}_4 = 22$. Combining the expression for ${\varvec{A}}$ with (29) and (32), we obtain

$$\begin{aligned} N_{eE} = \frac{1}{2(1-\max (1-1/(2N_1),B_{22}))} = \max \left( N_1,\frac{N_2}{2N_{ \text{ emig }}}\right) . \end{aligned}$$

For large enough migration rates $N_{ \text{ emig }}\ge N_2/(2N_1)$, the source population will determine the effective size, whereas for small migration rates $N_{ \text{ emig }}<N_2/(2N_1)$, the two populations get increasingly isolated, and the effective size tends to infinity. It turns out that Theorem 1 applies with $n=5$ components, of which the three non-absorbing ones are

$$\begin{aligned} \mathcal{X}_3&= \{{\varvec{x}}=(x_1,x_2); \, x_1=0, x_2\ne 0\},\\ \mathcal{X}_4&= \{{\varvec{x}}=(x_1,x_2); \, x_1=1, x_2\ne 1\},\\ \mathcal{X}_5&= \{{\varvec{x}}=(x_1,x_2); \, 0<x_1,x_2<1\}. \end{aligned}$$

For large migration rates, the eigenvalue $\lambda$ of ${\varvec{P}}$ and ${\varvec{A}}$ is found within components $\mathcal{X}_5$ and $\mathcal{C}_1$, and (42) simplifies to $\phi _3({\varvec{x}})=\rho _{11}x_1(1-x_1)$. For small migration rates, we find $\lambda$ within $\mathcal{X}_3,\mathcal{X}_4$ and $\mathcal{C}_2,\mathcal{C}_3$ respectively. $\square$

#### *Example 7*

*(Combined age and spatial structure)*. Age structured models have been studied by Felsenstein (1971), Hill (1972), Kaj et al. (2001), Sagitov and Jagers (2005) and Hössjer (2011). Here we consider a population with $s=2z$ that has two demes with $z$ age classes each. Subpopulations are numbered so that $i$ ($z+i$) corresponds to age class $i=1,\ldots ,z$ of deme 1 (deme 2). The expected forward and backward migration matrices

57 $$\begin{aligned} {\varvec{M}}= \left( \begin{array}{c@{\quad }c} {\varvec{M}}_{11} &{} {\varvec{M}}_{12} \\ {\varvec{M}}_{21} &{} {\varvec{M}}_{22} \end{array}\right) , \quad {\varvec{B}}= \left( \begin{array}{c@{\quad }c} {\varvec{B}}_{11} &{} {\varvec{B}}_{12} \\ {\varvec{B}}_{21} &{} {\varvec{B}}_{22} \end{array}\right) , \end{aligned}$$

have a block structure, with ${\varvec{M}}_{cd}$ and ${\varvec{B}}_{cd}$ describing migration between demes $c$ and $d$. All blocks of the forward matrix have the same form, e.g.

58 $$\begin{aligned} {\varvec{M}}_{11} = \left( \begin{array}{c@{\quad }c@{\quad }c@{\quad }c@{\quad }c@{\quad }c} M_{11} &{} M_{12} &{} 0 &{} \ldots &{} 0 &{} 0 \\ M_{21} &{} 0 &{} M_{2,3} &{} \ldots &{} 0 &{} 0 \\ \vdots &{} \vdots &{} \vdots &{} \ddots &{} \vdots &{} \vdots \\ M_{z-1,1} &{} 0 &{} 0 &{} \ldots &{} 0 &{} M_{z-1,z} \\ M_{z,1} &{} 0 &{} 0 &{} \ldots &{} 0 &{} 0 \\ \end{array} \right) , \end{aligned}$$

with a first column containing expected number of offspring in deme 1 of all age classes in deme 1, and a superdiagonal with probabilities of surviving to the next age class *and* not migrating to deme 2. The blocks of the backward matrix a similar structure, for instance

59 $$\begin{aligned} {\varvec{B}}_{11} = \left( \begin{array}{c@{\quad }c@{\quad }c@{\quad }c} B_{11} &{} \ldots &{} B_{1,z-1} &{} B_{1z}\\ B_{21} &{} \ldots &{} 0 &{} 0\\ \vdots &{} \ddots &{} \vdots &{} \vdots \\ 0 &{} \ldots &{} B_{z,z-1} &{} 0 \end{array}\right) , \end{aligned}$$

with probabilities in the first row that the parent of a newborn in deme 1 originates from the various age classes in deme 1, and probabilities along the subdiagonal that genes of the various adult age classes in deme 1 did not migrate during the last time step. If parental subpopulations are chosen independently for all genes from ${\varvec{B}}$, it follows that $Q_{ij,kl}$ is given by (51).

Coalescence probabilities

60 $$\begin{aligned} p_{ijk} = \left\{ \begin{array}{ll} 1/(2N_{eI,k}), &{} a(i)=a(j)=1,\\ 0, &{} a(i)=a(j)=a(k)+1,\\ 1/(2N_k), &{} a(i)=1,a(j)=a(k)+1 \text{ or } \\ &{} a(i)=a(k)+1,a(j)=1, \end{array}\right. \end{aligned}$$

are obtained for all triples $ijk$ of age classes for which $Q_{ij,kk}$ is nonzero, with $a(i)=i \text{ mod } z$ the age class of subpopulation $i$, assuming in the first row that parents of newborns in a particular deme are chosen from a mixed multinomial distribution, with coalescence probabilities as in (53). For the second row we used that the coalescence probability (54) for survival is zero, and in third row we assumed that survival is independent of number of offspring, cf. (55).

We finally obtain $N_{eE}$ by inserting (57) and (59) into (51), and then (51) and (60) into (41) and (32). $\square$

#### *Example 8*

*(Extended Moran model)*. Eldon and Wakeley (2006, 2009) extended the Moran model to allow for more skewed offspring distributions, for a homogeneous population and for the island model. Here generalize their model to populations with conservative migration. The reproduction cycle between time points $t-1$ and $t$ is divided into two parts. In the first reproduction step one gene within each subpopulation $k$ at time $t-1$ is chosen randomly to have $Y_{t-1,k}=Y_k$ offspring, including itself, where $2\le Y_k\le 2N_k$. Then $Y_k-1$ other, randomly chosen genes from the same subpopulation die. In the second migration step we assume that (44) holds, so that the forward and backward migration rates are non-random and fixed. The conservative migration assumption implies that exactly $2N_k$ genes from subpopulation $k$ migrate in (randomly chosen) groups of sizes $2N_1B_{1k},\ldots 2N_sB_{sk}$ to subpopulations $1,\ldots ,s$. The coalescence probability for any triple $i,j,k$ of subpopulations is

61 $$\begin{aligned} p_{ijk} = p_k = \sum _{y=2}^{2N_k} P(Y_k=y) \frac{{y\atopwithdelims ()2}{2N_k-y\atopwithdelims ()0}}{{2N_k \atopwithdelims ()2}} = \frac{E\left( Y_k(Y_k-1)\right) }{2N_k(2N_k-1)}, \end{aligned}$$

as shown either by a direct argument, or from (38), (39) and (45). We finally obtain $N_{eE}$ by inserting (45) and (61) into (41) and (32). $\square$

#### *Example 9*

*(Dioecious population)*. Consider a population with $N_m$ males, $N_f=N-N_m$ females and sex ratio $\xi =N_m/N$. Inheritance at an autosomal locus is modeled with $s=4$ subpopulations; gametes within males inherited from the father ($i=1$) and mother ($i=2$), and gametes within females inherited from the father ($i=3$) and mother ($i=4$), so that the relative subpopulation sizes are ${\varvec{u}}= \left( \xi /2,\xi /2,(1-\xi )/2,(1-\xi )/2\right)$. According to Mendelian laws, each gamete is inherited, with equal probability 0.5, either from a grandpaternal or a grandmaternal gamete within the father or mother. This gives an observed backward migration matrix $\varvec{\mathcal {B}}_t$ with a multinomial distribution (48). In view of (7), the expected backward/forward migration matrices are

62 $$\begin{aligned} {\varvec{B}}= \left( \begin{array}{c@{\quad }c@{\quad }c@{\quad }c} \frac{1}{2} &{} \frac{1}{2} &{} 0 &{} 0\\ 0 &{} 0 &{} \frac{1}{2} &{} \frac{1}{2} \\ \frac{1}{2} &{} \frac{1}{2} &{} 0 &{} 0\\ 0 &{} 0 &{} \frac{1}{2} &{} \frac{1}{2} \end{array}\right) , \quad {\varvec{M}}= \left( \begin{array}{c@{\quad }c@{\quad }c@{\quad }c} \frac{1}{2} &{} 0 &{} \frac{1-\xi }{2\xi }&{} 0\\ \frac{1}{2} &{} 0 &{} \frac{1-\xi }{2\xi }&{} 0\\ 0 &{} \frac{\xi }{2(1-\xi )}&{} 0 &{} \frac{1}{2}\\ 0 &{} \frac{\xi }{2(1-\xi )}&{} 0 &{} \frac{1}{2} \end{array}\right) , \end{aligned}$$

and ${\varvec{\gamma }}= (1/4,1/4,1/4,1/4)$ follows from (4). Pairwise backward migration probabilities $Q_{ij,kl}$ are given by (51), and in order to find the coalescence probabilities $p_{ijk}$, we follow the notation of Hill (1979) and let $m$ and $f$ represent male and female sexes, write $\tau _{rs}^2$ for the variance of the number of children of sex $s$ of an individual of sex $r$, and $\tau _{rr,rs}$ for the covariance of the number of children of sex $r$ and $s$ of an individual of sex $r$. It is shown in the “Appendix” that the nonzero coalescence probabilities when $k=1$ are

63 $$\begin{aligned} p_{111}&= \frac{\tau _{mm}^2}{2Nu_1-1},\end{aligned}$$

64 $$\begin{aligned} p_{331}&= \frac{1+\frac{\xi ^2}{(1-\xi )^2}\left( \tau _{mf}^2 -\frac{1-\xi }{\xi }\right) }{2Nu_1-\xi /(1-\xi )},\end{aligned}$$

65 $$\begin{aligned} p_{131}&= \frac{1+ \frac{\xi }{1-\xi }\tau _{mm,mf}}{2Nu_1}, \end{aligned}$$

and analogous formulas hold for $k=2,3,4$. In particular, if a sperm or ova gamete chooses its parent randomly among all $N_m$ or $N_f$ parental genes, the number of male and female offspring of a male are two independent binomial random variables $\text{ Bin }(2Nu_1,(2Nu_1)^{-1})$ and $\text{ Bin }(2Nu_3,(2Nu_1)^{-1})$, with variances $\tau _{mm}^2$ and $\tau _{mf}^2$ respectively, and covariance $\tau _{mm,mf}=0$. It is easily seen that in this case, all three probabilities in (63)–(65) equal $1/(2Nu_1)$. We finally obtain $N_{eE}$ by inserting (62) into (51), and then (51), (63)–(65) and the analogous coalescence probabilities for $k=2,3,4$ into (41) and (32). $\square$

## Asymptotics

According to (32), we find $N_{eE}$ from the largest eigenvalue $\lambda$ of ${\varvec{A}}$, for which we derived very explicit expressions in Sect. 4. Here will use this approach and give asymptotic formulas for $N_{eE}$ when

66 $$\begin{aligned} {\varvec{A}}={\varvec{A}}(\varepsilon ) = \left( A_{ij,kl}(\varepsilon )\right) _{ij,kl\in \mathcal{I}_2} \end{aligned}$$

depends on a positive parameter $\varepsilon \rightarrow 0$, with a Taylor expansion

67 $$\begin{aligned} {\varvec{A}}(\varepsilon ) = {\varvec{A}}(0) + \dot{{\varvec{A}}}\varepsilon + o(\varepsilon ) \text{ as } \varepsilon \rightarrow 0, \end{aligned}$$

and $\dot{{\varvec{A}}}=(\dot{A}_{ij,kl})_{ij\in \mathcal{I}_2,kl\in \mathcal{I}_2}$ a matrix of order $s^2$. For each fixed $\varepsilon >0$, ${\varvec{A}}(\varepsilon )$ satisfies the conditions of Theorem 2, so that in particular its unique largest eigenvalue is $\lambda =\lambda (\varepsilon )$. The limiting matrix ${\varvec{A}}(0)$ is assumed to have a largest, not necessarily unique, eigenvalue $\lambda (0)=1$. As in Maruyama (1970a) and Nagylaki (1980, 1995), we use perturbation theory of matrices (Horn and Johnson 1985; Friswell 1996), in order to deduce

68 $$\begin{aligned} \lambda (\varepsilon ) = 1 + \dot{\lambda }\varepsilon + o(\varepsilon ) \text{ as } \varepsilon \rightarrow 0, \end{aligned}$$

for some negative constant $\dot{\lambda }< 0$. Inserting (68) into (21), we find the asymptotic rate

69 $$\begin{aligned} N_{eE}(\varepsilon ) = \frac{1}{-2\dot{\lambda }\varepsilon } + o(\varepsilon ^{-1}) \text{ as } \varepsilon \rightarrow 0 \end{aligned}$$

by which the eigenvalue effective size tends to infinity. The following result gives a very explicit formula for $\dot{\lambda }$, see for instance Aa et al. (2007) and references therein for a proof:

### **Theorem 4**

Suppose the above conditions hold, with ${\varvec{A}}(0)$ having a largest eigenvalue $\lambda (0)=1$ of multiplicity $1\le v \le s^2$, with corresponding left and right eigenvectors ${\varvec{\rho }}_1(0),\ldots ,{\varvec{\rho }}_v(0)$ and ${\varvec{r}}_1(0),\ldots ,{\varvec{r}}_v(0)$. If also the perturbed left eigenvectors ${\varvec{\rho }}_\alpha (\varepsilon )$ and eigenvalues $\lambda _\alpha (\varepsilon )$ are differentiable functions of $\varepsilon$ at 0 for $\alpha =1,\ldots ,v$, it holds that $\lambda (\varepsilon ) = \max _{\alpha =1,\ldots ,v} \lambda _\alpha (\varepsilon )$ for small enough $\varepsilon >0$, and (68) is satisfied, with

70 $$\begin{aligned} \dot{\lambda }= \lambda _{ \text{ max }}(\dot{{\varvec{\Lambda }}}) \end{aligned}$$

and $\dot{{\varvec{\Lambda }}}=(\dot{\Lambda }_{\alpha \beta })$ a $v\times v$ matrix with entries

71 $$\begin{aligned} \dot{\Lambda }_{\alpha \beta } = {\varvec{\rho }}_\alpha (0)\dot{{\varvec{A}}}{\varvec{r}}_\beta (0). \end{aligned}$$

In particular, suppose ${\varvec{A}}(0)$ is the transition matrix of a Markov chain with a unique equilibrium distribution ${\varvec{\rho }}(0)=(\rho _{ij}(0))$, the left eigenvector corresponding to $\lambda (0)=1$, and a right eigenvector ${\varvec{r}}(0)={\varvec{1}}$. Then $v=1$ and

72 $$\begin{aligned} \dot{\lambda }= \sum _{ij,kl} \rho _{ij}(0)\dot{A}_{ij,kl}. \end{aligned}$$

In the following three subsections, the small perturbation parameter $\varepsilon$ will either correspond to an inverse population size, a migration rate or both. We will use (41) and establish a Taylor expansion (67) for each case based on

73 $$\begin{aligned} A_{ij,kl}(\varepsilon ) = \left( 1-\frac{1}{2N_i(\varepsilon )}\right) ^{\{i=j\}} \left( \frac{1-p_{ijk}(\varepsilon )}{1-\frac{1}{2N_k(\varepsilon )}}\right) ^{\{k=l\}} Q_{ij,kl}(\varepsilon ), \end{aligned}$$

when population sizes $N_i(\varepsilon )=N(\varepsilon )u_i(\varepsilon )$, backward migration rates $Q_{ij,kl}(\varepsilon )$ and/or coalescence probabilities $p_{ijk}(\varepsilon )$ depend on the perturbation parameter $\varepsilon \rightarrow 0$. The asymptotic expression for $N_{eE}$ is then obtained from (69) and (70).

### Large populations

We assume that the population size $N$ tends to infinity, while the relative subpopulations sizes ${\varvec{u}}$, forward and backward migration matrices ${\varvec{M}}$ and ${\varvec{B}}$ are kept fixed. Introduce

74 $$\begin{aligned} \varepsilon = \frac{1}{2N^\beta } \end{aligned}$$

as a perturbation parameter, with $0<\beta \le 1$ a fixed constant. In order to verify a Taylor expansion of ${\varvec{A}}(\varepsilon )$ in (73), we first consider the backward migration matrix ${\varvec{Q}}(\varepsilon )=(Q_{ij,kl}(\varepsilon )) = E(\varvec{\mathcal {Q}}_t(\varepsilon ))$ for pairs of genes. It does not depend on $\varepsilon$ for Dirichlet multinomial backward migration in (50), whereas for fixed backward migration (45) it does. In order to keep generality we assume that ${\varvec{Q}}(\varepsilon )$ may depend on $\varepsilon$, with a Taylor expansion

75 $$\begin{aligned} {\varvec{Q}}(\varepsilon ) = {\varvec{Q}}(0) + \dot{{\varvec{Q}}}\varepsilon + o(\varepsilon ) \text{ as } \varepsilon \rightarrow 0 \end{aligned}$$

for some matrix $\dot{{\varvec{Q}}}= (\dot{Q}_{ij,kl})$. It will be seen though that $\dot{{\varvec{Q}}}$ does not influence the asymptotic behavior of $N_{eE}$. In contrast, the asymptotic behavior of the coalescence probabilities $p_{ijk}=p_{ijk}(\varepsilon )$ is crucial and depends on how variable reproductive success is between individuals that migrate from one subpopulation ($k$) to other pairs of subpopulations ($i,j$). The limits

76 $$\begin{aligned} \sigma _{ijk} = \lim _{N\rightarrow \infty } \frac{V_{kij}u_k^2}{(Nu_k)^{1 -\beta }u_iu_jQ_{ij,kk}(0)} \end{aligned}$$

are assumed to exist for all triples $ijk$, with $V_{kij}$ defined in (38). It follows from (39), (74) and (76) that the coalescence probabilities admit Taylor expansions

77 $$\begin{aligned} p_{ijk}(\varepsilon ) = \frac{\sigma _{ijk}}{u_k^\beta }\varepsilon + o(\varepsilon ) := \dot{p}_{ijk}\varepsilon + o(\varepsilon ) \text{ as } \varepsilon \rightarrow 0 \end{aligned}$$

for all $i,j,k$. We refer to $\dot{p}_{ijk}$ as the coalescence rate when two lines from $i$ and $j$ are merged into $k$ and time is measured in units of $\varepsilon ^{-1}=2N^\beta$ generations. The coalescence rate $\sigma _{ijk}$ takes the size of the parental subpopulation $k$ into account and measures time in units of $2(Nu_k)^\beta$ instead. Taking the $\varepsilon \rightarrow 0$ limit in (73), it follows from (75) and (77) that

78 $$\begin{aligned} {\varvec{A}}(0) = {\varvec{Q}}(0). \end{aligned}$$

We assume that ${\varvec{Q}}(0)$ is the transition kernel of a Markov chain that is not necessarily irreducible (it may contain some transient states), but has a unique equilibrium distribution ${\varvec{\rho }}(0)=(\rho _{ij}(0))$, which is also the left eigenvector of ${\varvec{Q}}(0)$ corresponding to its unique largest eigenvalue $\lambda (0)=1$. Hence formula (72) of Theorem 4 applies, and we obtain the following:

#### **Theorem 5**

Define $\varepsilon$ and ${\varvec{A}}={\varvec{A}}(\varepsilon )$ as in (73)–(74). Assume the population size $N\rightarrow \infty$ so that (75) holds and the limits in (76) exist for some $0<\beta \le 1$. Then ${\varvec{A}}(\varepsilon )$ satisfies Taylor expansion (67), with ${\varvec{A}}(0)$ as in (78) and

79 $$\begin{aligned} \dot{A}_{ij,kl}&= -1_{\{k=l\}}\frac{\sigma _{ijk}Q_{ij,kk}(0)}{u_k^\beta } + \dot{Q}_{ij,kl}\nonumber \\&+ 1_{\{\beta =1\}}\left( 1_{\{k=l\}}\frac{Q_{ij,kk}(0)}{u_k}- 1_{\{i=j\}} \frac{Q_{ii,kl}(0)}{u_i}\right) . \end{aligned}$$

If the differentiability conditions on $\lambda (\cdot )$ and ${\varvec{\rho }}(\cdot )$ in Theorem 4 hold, then

80 $$\begin{aligned} N_{eE} = \frac{N^\beta }{C} + o(N^\beta ) \text{ as } N\rightarrow \infty , \end{aligned}$$

with

81 $$\begin{aligned} C = \sum _{ijk} \frac{\rho _{ij}(0)\sigma _{ijk}Q_{ijkk}(0)}{u_k^\beta }. \end{aligned}$$

Suppose $I=I_\tau$ and $J=J_\tau$ are the subpopulations of the ancestors, taken from the same generation $t-\tau$ of two genes sampled at a fixed time $t$. Let $K=I_{\tau +1}$ and $L=J_{\tau +1}$ refer to the subpopulations of their two parents. Arguing as in Möhle (1998a), it will take many generations before coalescence in a large population, so that $(I,J,K,L)$ will first attain its equilibrium distribution ${\varvec{\rho }}_{ij}(0)Q_{ij,kl}(0)$. Therefore, (81) can be interpreted as a coalescence rate

82 $$\begin{aligned} C = E\left( 1_{\{K=L\}} \dot{p}_{IJK}\right) = E\left( 1_{\{K=L\}} \frac{\sigma _{IJK}}{u_K^\beta }\right) \end{aligned}$$

at equilibrium if time is counted in units of $2N^\beta$. In view of this, we may expect that for large populations, $N_{eE}$ is asymptotically equivalent to the coalescence effective size $N_{eC}$ whenever the latter exists. In a number of examples below, we will indeed verify that

83 $$\begin{aligned} N_{eC} = \frac{N^\beta }{C}, \end{aligned}$$

with $C$ the same constant as in (81). To this end, we first need the following:

#### **Corollary 4**

Suppose the conditions of Theorem 5 hold. Then asymptotically as $N\rightarrow \infty$, $N_{eE}$ is given by (80)–(81), with

84 $$\begin{aligned} Q_{ij,kl}(0) = B_{ik}B_{jl}, \end{aligned}$$

for fixed backward migration (44), and

85 $$\begin{aligned} Q_{ij,kl}(0) = B_{ik}B_{jl} + 1_{\{i=j\}}\frac{1_{\{k=l\}} B_{ik}-B_{ik}B_{jl}}{\alpha _i+1}, \end{aligned}$$

for Dirichlet multinomial backward migration (46)–(47).

Theorem 2 and (85) imply that ${\varvec{A}}$ has $m=1$ irreducible component $\mathcal{C}_1=\mathcal{I}_2$ for Dirichlet multinomial backward migration when ${\varvec{B}}$ is irreducible and at least one $\alpha _i>0$, whereas ${\varvec{A}}$ has $m=2$ components $\mathcal{C}_1 = \{(i,i); \, i=1,\ldots ,s\}$ and $\mathcal{C}_2 = \{(i,i); \, i\ne j\}$ when $\alpha _i\equiv 0$. Then the joint ancestry of two genes are confined to lie in the same subpopulation after a few generations, once their ancestral subpopulation lineages merge for the first time, although they may not yet have coalesced at the gene level.

#### **Corollary 5**

Assume the conditions of Theorem 5 hold, with Dirichlet multinomial backward migration and $\alpha _i\equiv 0$. Then the equilibrium distribution ${\varvec{\rho }}(0)=(\rho _{ij}(0))$ of ${\varvec{Q}}(0)$ is supported on the diagonal of $\mathcal{I}_2$, with elements

86 $$\begin{aligned} \rho _{ij}(0)=1_{\{i=j\}}\gamma _i. \end{aligned}$$

Moreover, $N_{eE}$ is asymptotically given by (80), with

87 $$\begin{aligned} C&= \sum _{ik} \frac{\gamma _i\sigma _{iik}B_{ik}}{u_k^\beta },\end{aligned}$$

88 $$\begin{aligned} \sigma _{iik}&= \lim _{N\rightarrow \infty } \frac{\bar{V}_{kii} u_k^2}{(Nu_k)^{1-\beta }u_i^2}, \end{aligned}$$

$\bar{V}_{kii} = E\left( \nu _{tki1}(\nu _{tki1}-1)|K_{ti}=k\right)$ and $K_{ti}$ the subpopulation to which all parents of the genes in $i$ at time $t$ belong.

Jagers and Sagitov (2004) and Pollak (2010) studied populations with rapidly varying sizes, which can be viewed as a special case of the Dirichlet multinomial backward distribution with $\alpha _i\equiv 0$ (see Example 2). They showed that $N_{eC}$ satisfies (83), with $\beta =1$ and $C$ as in (87).

It is also possible to get explicit expressions for $C$ when backward migration is fixed or multinomial:

#### **Corollary 6**

Assume the conditions of Theorem 5 hold, with fixed or multinomial ($\alpha _i\equiv \infty$) backward migration. Then $Q_{ij,kl}(0)=B_{ik}B_{jl}$ follows from (84) and (85) with $\alpha _i\equiv \infty$ respectively, and the equilibrium distribution ${\varvec{\rho }}(0)=(\rho _{ij}(0))$ has elements

89 $$\begin{aligned} \rho _{ij}(0) = \gamma _i\gamma _j. \end{aligned}$$

Moreover, $N_{eE}$ is asymptotically given by (80)–(81), with

90 $$\begin{aligned} C&= \sum _{ijk}\frac{\gamma _i\gamma _j\sigma _{ijk}B_{ik}B_{jk}}{u_k^\beta },\end{aligned}$$

91 $$\begin{aligned} \sigma _{ijk}&= \lim _{N\rightarrow \infty }\frac{V_{kij}}{(Nu_k)^{1-\beta }M_{ki}M_{kj}}, \end{aligned}$$

and $V_{kij}$ as defined in (38).

Felsenstein (1971) seems to have been first to use (89) for weighting pairs of subpopulations. Hössjer (2011) studied models with fixed backward migration, and showed that $N_{eC}$ satisfies (83) when $\beta =1$, with $C$ as in (90).

#### *Example 10*

*(Local subpopulation sizes)*. When the coalescence probability $p_{ijk}(\varepsilon )=p_k(\varepsilon )$ only depends on the parental subpopulation $k$ for all $\varepsilon >0$, the size standardized coalescence rate (76) satisfies

92 $$\begin{aligned} \sigma _{ijk} = \sigma _k, \end{aligned}$$

as for the mixed multinomial reproduction scheme of Example 3. We deduce from (53) that $p_{k}\ge 1/(2Nu_k)$, and

93 $$\begin{aligned} \sigma _{ijk} = \sigma _k = 1_{\{\beta =1\}} + 4 \lim _{N\rightarrow \infty } \left( (Nu_k)^{1+\beta } \text{ Var }(\omega _{tk1})\right) , \end{aligned}$$

with $C$ larger and $N_{eE}$ smaller the more variable the gene weights $\omega _{tkg}$ are. When (92) and Theorem 5 hold, it follows that $N_{eE}$ is given by (80), with

94 $$\begin{aligned} C&= \sum _{k} \frac{\sigma _k}{u_k^\beta } \sum _{ij} \rho _{ij}(0) Q_{ijkk}(0)\nonumber \\&= \sum _{k=1}^s \frac{\sigma _k\rho _{kk}(0)}{u_k^\beta }, \end{aligned}$$

using $\sum _{ij} \rho _{ij}(0)Q_{ij,kl}(0) = \rho _{kl}(0)$ in the last step, since ${\varvec{\rho }}(0)$ is the left eigenvector of ${\varvec{Q}}(0)$ with eigenvalue 1. In particular, if all genes within each subpopulation have parents from the same subpopulation, (86) implies

95 $$\begin{aligned} C = \sum _{k=1}^s \frac{\sigma _k\gamma _k}{u_k^\beta }, \end{aligned}$$

whereas for fixed or multinomial backward migration, we deduce

96 $$\begin{aligned} C = \sum _{k=1}^s \frac{\sigma _k\gamma _k^2}{u_k^\beta } \end{aligned}$$

from (89). In particular, if offspring pick their parents uniformly and independently within the parental subpopulation $k$, we have $p_{ijk} = p_k = 1/(2N_k)$, so that the local inbreeding effective size $N_{eI,k}$ in (53) equals the local census size $N_k$. Asymptotically, this corresponds to having $\beta =1$ in (74) and $\sigma _k=1$ in (92). For fixed backward migration, we can therefore use (80) and (96), and deduce $N_{eE}=N/C + o(N)$ as $N\rightarrow \infty$, with

97 $$\begin{aligned} C = \sum _{k=1}^s \frac{\gamma _k^2}{u_k}. \end{aligned}$$

Notohara (1993) and Nordborg and Krone (2002) showed that the coalescence effective size satisfies $N_{eC}=N/C$, with $C$ as in (97). Whenever $N_{eI,k}=N_k$,

98 $$\begin{aligned} \sum _{k=1}^s \frac{\gamma _k}{u_k}&= C(\alpha _i\equiv 0) \ge \nonumber \\ \sum _{k=1}^s \frac{\gamma _k^2}{u_k}&= C(\alpha _i\equiv \infty )\ge \nonumber \\ 1&= C(\alpha _i\equiv \infty ;\gamma _k=u_k). \end{aligned}$$

The first inequality of (98) shows how much stochastically varying migration lowers $N_{eE}$ at most. Then Cauchy-Schwarz inequality shows how much a variable long term reproductivity $\gamma _k/u_k$ between subpopulations lowers $N_{eE}$, with equality for conservative migration $\gamma _k=u_k$ (Nagylaki 1980). The circular stepping stone model has conservative migration with uniform population sizes $u_k=1/s$, and Maruyama (1970a) found that $N_{eE}/N\rightarrow 1$ as $N\rightarrow \infty$, in agreement with the right hand side of (98). $\square$

#### *Example 11*

*(Multiple mergers)*. Other limiting ancestral processes with multiple mergers (Pitman 1999; Sagitov 1999) are possible. Let $p_{ijh,k}^{(3)}$ be the probability that three genes from subpopulations $i,j,h$ that all have their parents in subpopulation $k$, have the same parent. We will only consider models for which the pairwise and triple coalescence probabilities $p_{ijk}=p_k$ and $p_{ijh,k}^{(3)}=p_k^{(3)}$ only depend on the source population. Then we must have

99 $$\begin{aligned} \frac{p_k^{(3)}(\varepsilon )}{p_k(\varepsilon )} \rightarrow 0 \text{ as } \varepsilon \rightarrow 0 \end{aligned}$$

in order for the limiting process to be Kingman’s coalescent, see Theorem 3.2 of Möhle (2000). It is not possible to violate (99) in Example 3. Indeed,

$$\begin{aligned} \frac{p_k^{(3)}}{p_k} = \frac{2Nu_k E(\omega _{tk1}^3)}{2Nu_k E(\omega _{tk1}^2)} = \frac{E(\omega _{tk1}^3)}{E(\omega _{tk1}^2)} \rightarrow 0, \end{aligned}$$

as $N\rightarrow \infty$, since $\omega _{tk1}$ is bounded by 1 and tends to zero in probability. On the other hand, the Moran model of Example 8 allows for multiple mergers for an appropriate choice of the offspring size $Y_{t-1,k}=Y_k$ of the reproducing gene of subpopulation $k$. As in Eldon and Wakeley (2006), we let

$$\begin{aligned} P(Y_k=y) = \left\{ \begin{array}{l@{\quad }l} 1 - (2N_k)^{-\beta }, &{} y=2,\\ (2N_k)^{-\beta }, &{} y = 2N_k\psi ,\\ 0, &{} \text{ otherwise }, \end{array}\right. \end{aligned}$$

for some $0<\psi \le 1$ and $\beta > 0$. Then one shows

$$\begin{aligned} p^{(3)}_k=\frac{E\left( Y_k(Y_k-1)(Y_k-2)\right) }{2N_k(2N_k-1)(2N_k-2)} \end{aligned}$$

analogously as in (61). Since

$$\begin{aligned} E\left( Y_k(Y_k-1)\right)&\sim \psi ^2 (2N_k)^{2-\beta } + 2,\\ E\left( Y_k(Y_k-1)(Y_k-2)\right)&\sim \psi ^3 (2N_k)^{3-\beta }, \end{aligned}$$

as $N\rightarrow \infty$, it follows from (61) and the last two displayed equations that we can violate (99) when $0<\beta \le 1$, with $\sigma _{ijk} = \sigma _k = \psi ^2 2^{1-\beta }$. Since the extended Moran model has fixed backward migration and conservative migration $\gamma _k=u_k$, it follows that $N_{eE}$ satisfies (96) with

$$\begin{aligned} C = 2^{1-\beta }\psi ^2 \sum _{k=1}^s u_k^{2-\beta }. \end{aligned}$$

Notice that this expression equals 1 when $\beta =\psi =1$, since then the coalescence probability is asymptotically equivalent to $1/(2N_k)$. $\square$

Other applications of Theorem 5 with $\beta =1$ includes a single deme with $s$ age classes. Explicit formulas for the constant $C$ in $N_{eE}=N/C + o(N)$ can be found under general assumptions on how reproductivity varies randomly between and within age classes, thereby extending results of Felsenstein (1971), Sagitov and Jagers (2005) and Hössjer (2011).

For dioecious models, Hill (1979) found that $N_{eE}=N/C + o(N)$, with

100 $$\begin{aligned} C&= \frac{1}{16\xi }\left( 2 + \tau _{mm}^2 + 2\frac{\xi }{1-\xi } \tau _{mm,mf} + \left( \frac{\xi }{1-\xi }\right) ^2\tau _{mf}^2 \right) \nonumber \\&+\frac{1}{16(1-\xi )}\left( 2 + \tau _{ff}^2 + 2\frac{1-\xi }{\xi } \tau _{ff,fm} + \left( \frac{1-\xi }{\xi }\right) ^2\tau _{fm}^2 \right) , \end{aligned}$$

which also follows from (51), (62), (63)–(65) and (90). Other effective size expressions of a diploid population can be found in Crow and Denniston (1988), Caballero (1995) and Nagylaki (1995). The latter two authors also treat inheritance at sex-linked loci. The expression for $C$ is then somewhat different, since males only have one copy of an $X$-chromosome, and only $s=3$ subpopulations are needed. Overlapping generations within a dioecious population (Pollak 2011) requires $s=4z$ ($s=3z$) subpopulations for inheritance at an autosomal (X-linked) locus with $z$ age classes. See also Möhle (1998b), for coalescence theory of two-sex models.

### Small migration rates

Assume that the subpopulations in (1) divide into $m\le s$ demes

101 $$\begin{aligned} \mathcal{I}= \mathcal{I}(1) \cup \cdots \cup \mathcal{I}(m), \end{aligned}$$

with deme $d$ containing the subpopulations of $\mathcal{I}(d)$. We will introduce a migration parameter $\varepsilon \rightarrow 0$ that quantifies the amount of migration between the demes (not within them) while the total population size $N$ is kept fixed. In order to obtain an expression for $N_{eE}$ as $\varepsilon \rightarrow 0$, the crucial part is to find how all $Q_{ij,kl}(\varepsilon )$ in (73) depend on $\varepsilon$. Although the relative subpopulation sizes $u_i(\varepsilon )$ and coalescence probabilities $p_{ijk}(\varepsilon )$ may vary with $\varepsilon$ to some extent, this will have no asymptotic impact on $N_{eE}$ as $\varepsilon \rightarrow 0$.

We will assume that the backward migration matrix

102 $$\begin{aligned} {\varvec{B}}(\varepsilon ) = {\varvec{B}}(0) + \varepsilon \dot{{\varvec{B}}}\end{aligned}$$

depends on $0\le \varepsilon \le \varepsilon _{ \text{ max }}$, where $\varepsilon _{ \text{ max }}$ is chosen to guarantee that ${\varvec{B}}(\varepsilon )$ remains a non-negative matrix. The demes are isolated when $\varepsilon =0$, so that ${\varvec{B}}(0)$ has a block diagonal structure ${\varvec{B}}(0) = \text{ diag }({\varvec{B}}_{11}(0),\ldots ,{\varvec{B}}_{mm}(0))$, with ${\varvec{B}}_{dd}(0)=(B_{ik}(0))_{i,k\in \mathcal{I}(d)}$ describing backward migration within deme $d$. Since ${\varvec{B}}(\varepsilon )$ is a transition matrix of a Markov chain for all $\varepsilon$, the row sums of $\dot{{\varvec{B}}}$ must be zero, and this holds, for instance, if

103 $$\begin{aligned} \dot{B}_{ik} = 1_{\{k\notin \mathcal{I}(d)\}}\dot{B}_{ik} - 1_{\{k\in \mathcal{I}(d)\}}B_{ik}(0)\sum _{l\notin \mathcal{I}(d)}\dot{B}_{il} \end{aligned}$$

for all $i\in \mathcal{I}(d)$ and $d=1,\ldots ,m$. If ${\varvec{M}}(\varepsilon )$ and ${\varvec{u}}(\varepsilon )$ are computed for each $\varepsilon >0$ from (7) and (8), it follows from (102) that

104 $$\begin{aligned} {\varvec{M}}(\varepsilon ) = \varepsilon \dot{{\varvec{M}}}+ o(\varepsilon ), \end{aligned}$$

if all $u_i(\varepsilon )$ are differentiable at $0$, with $\dot{{\varvec{M}}}=(\dot{M}_{ki})$ having elements

105 $$\begin{aligned} \dot{M}_{ki}=u_i(0)\dot{B}_{ik}/u_k(0), \quad { when }\,k\in \mathcal{I}(a)\ne \mathcal{I}(b)\ni i. \end{aligned}$$

The migration parameter $\varepsilon$ is such $B(\varepsilon )=\dot{B}\varepsilon + o(\varepsilon )$ and $M(\varepsilon ) = \dot{M}\varepsilon + o(\varepsilon )$ as $\varepsilon \rightarrow 0$ for some positive constants $\dot{B}$ and $\dot{M}$, where

106 $$\begin{aligned} B(\varepsilon ) = \sum _{d=1}^m \sum _{i\in \mathcal{I}(d)} \gamma _i(\varepsilon ) \sum _{k;k\ne \mathcal{I}(d)} B_{ik}(\varepsilon ), \end{aligned}$$

is the backward migration rate between demes, i.e. the average number of parents of ancestors far back in time that originate from another deme than their children, and

107 $$\begin{aligned} M(\varepsilon ) = \sum _{d=1}^m \sum _{k\in \mathcal{I}(d)} u_k(\varepsilon ) \sum _{i;i\ne \mathcal{I}(d)} M_{ki}(\varepsilon ), \end{aligned}$$

is the forward migration rate, i.e. the fraction of all offspring today whose parents reside in another deme. Backward migration $B(\varepsilon )$ is somewhat easier to analyze theoretically, but often $\varvec{M}(\varepsilon )$ is of more interest in applications.

In order to find explicit expressions for $\dot{B}$ and $\dot{M}$, we introduce ${\varvec{\gamma }}_d = (\gamma _{di})_{i\in \mathcal{I}(d)}$ as the equilibrium distribution of ${\varvec{B}}_{dd}(0)$, and the matrix ${\varvec{G}}=(G_{ab})_{a,b=1}^m$ with elements

108 $$\begin{aligned} G_{ab} = \sum _{i\in \mathcal{I}(a)} \gamma _{ai}\sum _{k\in \mathcal{I}(b)} \dot{B}_{ik}. \end{aligned}$$

It is the infinitesimal generator of a continuous time Markov process with state space $\{1,\ldots ,m\}$ and an equilibrium distribution ${\varvec{\theta }}= (\theta _1,\ldots ,\theta _m)$ satisfying

109 $$\begin{aligned} {\varvec{\theta }}{\varvec{G}}&= {\varvec{0}},\nonumber \\ \sum _{d=1}^m \theta _d&= 1. \end{aligned}$$

In the next lemma we assume $\varepsilon$ is small, so that migration is faster within than between demes, and subpopulations within a deme form a macro state. The backward ancestry of a gene then attains its equilibrium distribution within a deme before any transitions between demes occur, and then the backward deme ancestry is a continuous time Markov process with generator ${\varvec{G}}$:

#### **Lemma 1**

Suppose $\varepsilon \rightarrow 0$. Then the equilibrium distribution ${\varvec{\gamma }}(\varepsilon )$ corresponding to (102) satisfies

110 $$\begin{aligned} \gamma _i(\varepsilon ) = 1_{\{i\in \mathcal{I}(d)\}}\theta _d \gamma _{di} + o(1), \end{aligned}$$

for $i=1,\ldots ,s$, and the backward migration rate (106)

111 $$\begin{aligned} B(\varepsilon ) = - \varepsilon \sum _{d=1}^m \sum _{i,k\in \mathcal{I}(d)} \gamma _i(0) \dot{B}_{ik} + o(\varepsilon ), \end{aligned}$$

where $\gamma _i(0)$ is the limit of the right hand side of (110). If all $u_i(\varepsilon )$ are differentiable at 0, the forward rate (107) has a similar expansion

112 $$\begin{aligned} M(\varepsilon ) = \varepsilon \sum _{d=1}^m \sum _{k\in \mathcal{I}(d)} u_k(0)\sum _{i\notin \mathcal{I}(d)} \dot{M}_{ki} + o(\varepsilon ). \end{aligned}$$

In order to find the asymptotic behaviour of $N_{eE}$ as $\varepsilon \rightarrow 0$ by means of (69) and Theorem 4, we derive an expression for ${\varvec{A}}(\varepsilon )$ in (73), find $\dot{{\varvec{A}}}$, show that ${\varvec{A}}(0)$ has a largest eigenvalue $\lambda (0)=1$, find its multiplicity $v$ and corresponding left and right eigenvectors. Because all demes are isolated when $\varepsilon =0$, it is easy to see that the ancestors of $i\in \mathcal{I}(a)$ and $j\in \mathcal{I}(b)$ must belong to $k\in \mathcal{I}(a)$ and $l\in \mathcal{I}(b)$ respectively. Therefore ${\varvec{Q}}(0)$ has a block diagonal structure

113 $$\begin{aligned} {\varvec{Q}}(0) = \text{ diag }\left( {\varvec{Q}}_{ab}(0); 1\le a,b \le m\right) \end{aligned}$$

with ${\varvec{Q}}_{ab}(0) = \left( Q_{ij,kl}\right) _{i, k\in \mathcal{I}(a), j,l\in \mathcal{I}(b)}$ a square matrix of order $|\mathcal{I}(a)||\mathcal{I}(b)|$ containing all backward transitions when one gene and its parent are from deme $a$ and the other gene and its parent are from deme $b$. It follows from (73) that ${\varvec{A}}(0)$ has a block diagonal structure

114 $$\begin{aligned} {\varvec{A}}(0) = \text{ diag }\left( {\varvec{A}}_{ab}(0); 1\le a,b \le m\right) \end{aligned}$$

as well, with ${\varvec{A}}_{ab}(\varepsilon ) = \left( A_{ij,kl} (\varepsilon )\right) _{i,k\in \mathcal{I}(a),j,l\in \mathcal{I}(b)}$ having elements

115 $$\begin{aligned} A_{ij,kl}(\varepsilon )&= Q_{ij,kl}(\varepsilon )\nonumber \\&= B_{ik}(\varepsilon )B_{jl}(\varepsilon ), \quad i,k\in \mathcal{I}(a), j,l\in \mathcal{I}(b), a\ne b, \end{aligned}$$

for any $\varepsilon$ because of (73), for subpopulations $i$ and $j$ that reside in different demes. In particular, ${\varvec{A}}_{ab}(0)={\varvec{Q}}_{ab}(0)$ has a unique largest eigenvalue 1 when $a\ne b$, and associated left and right eigenvectors ${\varvec{\rho }}_{ab}(0) = \text{ vec }\left( (\rho _{ab,ij} (0))_{ij\in \mathcal{I}_2}\right) ^\prime$ and ${\varvec{r}}_{ab}(0) = \text{ vec }\left( (r_{ab,ij}(0))_{ij\in \mathcal{I}_2}\right)$ with components

116 $$\begin{aligned} \rho _{ab,ij}(0)&= \gamma _{ai}\gamma _{bj}1_{\{i\in \mathcal{I}(a),j\in \mathcal{I}(b)\}},\nonumber \\ r_{ab,ij}(0)&= 1_{\{i\in \mathcal{I}(a),j\in \mathcal{I}(b)\}}. \end{aligned}$$

Since coalescence events are possible within each deme, even when $\varepsilon =0$, it follows that ${\varvec{A}}_{dd}(0)$ differs from ${\varvec{Q}}_{dd}(0)$, with a largest eigenvalue strictly smaller than one. Therefore, the largest eigenvalue 1 of ${\varvec{A}}(0)$ has multiplicity

$$\begin{aligned} v = |\{\alpha =(a,b); \,\, 1\le a\ne b\le m\}| = m(m-1). \end{aligned}$$

In order to apply Theorem 4 we must also find the entries of the matrix $\dot{{\varvec{\Lambda }}}=(\dot{\Lambda }_{\alpha , \beta })_{\alpha , \beta =1}^v$, where $\alpha =(a,b)$, $\beta =(c,d)$, $a\ne b$ and $c\ne d$. Suppose $i\in \mathcal{I}(a)$, $j\in \mathcal{I}(b)$, $k\in \mathcal{I}(c)$ and $l\in \mathcal{I}(d)$, then

117 $$\begin{aligned} \dot{A}_{ij,kl} = \dot{Q}_{ij,kl} = \left\{ \begin{array}{ll} B_{ik}(0)\dot{B}_{jl} + \dot{B}_{ik}B_{jl}(0), &{} c=a\ne b=d,\\ B_{ik}(0)\dot{B}_{jl}, &{} c=a\ne b \ne d,\\ \dot{B}_{ik}B_{jl}(0), &{} c\ne a \ne b=d,\\ 0, &{} c\ne a \ne b \ne d \end{array}\right. \end{aligned}$$

follows from (114) and differentiation of (115) with respect to $\varepsilon$. Invoking the definition of $\dot{{\varvec{\Lambda }}}$ in (71), we find that

118 $$\begin{aligned} \dot{\Lambda }_{ab,cd}&= {\varvec{\rho }}_{ab}(0)\dot{{\varvec{A}}}{\varvec{r}}_{cd}\nonumber \\&= \mathop {\sum }\nolimits _{\begin{array}{l}i,j\in \mathcal{I}(a)\times \mathcal{I}(b)\\ k,l\in \mathcal{I}(c)\times \mathcal{I}(d)\end{array}}\rho _{ab,ij}(0)\dot{A}_{ij,kl}\nonumber \\&= \mathop {\sum }\nolimits _{\begin{array}{l} i,j\in \mathcal{I}(a)\times \mathcal{I}(b)\\ k,l\in \mathcal{I}(c)\times \mathcal{I}(d)\\ \end{array}} \gamma _{ai}\gamma _{bj}\dot{A}_{ij,kl}. \end{aligned}$$

Then we insert (117) into (118), make use of (108) and obtain

119 $$\begin{aligned} \dot{\Lambda }_{ab,cd} = \left\{ \begin{array}{ll} G_{aa}+G_{bb}, &{} c=a\ne b=d,\\ G_{bd}, &{} c=a\ne b \ne d,\\ G_{ac}, &{} c\ne a \ne b=d,\\ 0, &{} c\ne a \ne b \ne d. \end{array}\right. \end{aligned}$$

In particular, when each subpopulation is a deme, $m=s$ and $\mathcal{I}(d) = \{d\}$ for $d=1,\ldots ,s$, so that (108) implies ${\varvec{G}}=\dot{{\varvec{B}}}$, and $\dot{{\varvec{\Lambda }}}=(\dot{\Lambda }_{ij,kl})_{1\le i\ne j\le s, 1\le k\ne l\le s}$ is of order $v=s(s-1)$, with elements

120 $$\begin{aligned} \dot{\Lambda }_{ij,kl} = \left\{ \begin{array}{ll} \dot{B}_{ii}+\dot{B}_{jj}, &{} k=i\ne j=l,\\ \dot{B}_{jl}, &{} k=i\ne j\ne l,\\ \dot{B}_{ik}, &{} k\ne i\ne j=l,\\ 0, &{} \text{ otherwise }. \end{array}\right. \end{aligned}$$

Combining (111), (112) and (119) with Theorem 4, we obtain the following:

#### **Theorem 6**

Suppose subpopulations are divided into $m$ demes, as in (101), whose isolation is quantified by the matrix $\dot{{\varvec{B}}}=(\dot{B}_{ik})$ in (102). Then

121 $$\begin{aligned} N_{eE} = \frac{\sum _{d=1}^m \sum _{i,k\in \mathcal{I}(d)} \gamma _i(0) \dot{B}_{ik}}{2\lambda _{ \text{ max }}(\dot{{\varvec{\Lambda }}})} \cdot \frac{1}{B} + o(B^{-1}) \text{ as } B\rightarrow 0, \end{aligned}$$

with $B=B(\varepsilon )$ the backward migration rate between demes in (106), and $\dot{{\varvec{\Lambda }}}=(\dot{\Lambda }_{ab,cd})$ the matrix in (119), which simplifies to (120) when $m=s$. If all $u_k(\varepsilon )$ are differentiable at $\varepsilon =0$ as well, then

122 $$\begin{aligned} N_{eE} = - \frac{\sum _{d=1}^m \sum _{k\in \mathcal{I}(d)} u_k(0)\sum _{i\notin \mathcal{I}(d)} \dot{M}_{ki}}{2\lambda _{ \text{ max }}(\dot{{\varvec{\Lambda }}})} \cdot \frac{1}{M} + o(M^{-1}) \text{ as } M\rightarrow 0, \end{aligned}$$

with $M=M(\varepsilon )$ the forward migration rate in (107) and $\dot{M}_{ki}$ defined in (105).

#### *Example 12*

*(Island model)*. The island model (Wright 1943; Maruyama 1970b) is the most well known example of a population with spatial substructure, having $m=s$ demes, and a forward migration matrix

123 $$\begin{aligned} {\varvec{M}}(\varepsilon ) = (1-\varepsilon ){\varvec{I}}+ \frac{\varepsilon }{s-1}({\varvec{1}}{\varvec{1}}^\prime - {\varvec{I}}), \end{aligned}$$

where ${\varvec{1}}$ is a column vector of $s$ ones. Migration is symmetric, so that the migration rate $M_{ki} = \varepsilon /(s-1)$ from each $k$ to any other deme $i\ne k$ is the same. It follows by symmetry from (4), (7) and (8) that ${\varvec{B}}= {\varvec{M}}$, $M=B=\varepsilon$ and $u_k(\varepsilon )=\gamma _k(\varepsilon )=1/s$. This implies in particular that

124 $$\begin{aligned} \dot{B}_{ik} = \dot{M}_{ki} = \left\{ \begin{array}{ll} -1, &{} k=i,\\ 1/(s-1), &{} k\ne i. \end{array}\right. \end{aligned}$$

Insertion of (124) into (120) yields

$$\begin{aligned} \dot{\Lambda }_{ij,kl} = \left\{ \begin{array}{ll} -2, &{} k=i\ne j=l,\\ 1/(s-1), &{} k=i\ne j\ne l,\\ 1/(s-1), &{} k\ne i\ne j=l,\\ 0, &{} \text{ otherwise }, \end{array}\right. \end{aligned}$$

so that by symmetry, the largest eigenvalue of $\dot{{\varvec{\Lambda }}}$ corresponds to an eigenvector ${\varvec{1}}_v = (1,\ldots ,1)^\prime$ that is a column vector with $v=s(s-1)$ ones. Hence we find, from any of the row sums of $\dot{{\varvec{\Lambda }}}$, that

$$\begin{aligned} \lambda _{ \text{ max }}(\dot{{\varvec{\Lambda }}}) = - 2 + (s-2)\cdot \frac{1}{s-1} + (s-2)\cdot \frac{1}{s-1} = - \frac{2}{s-1}. \end{aligned}$$

**Fig. 1:**
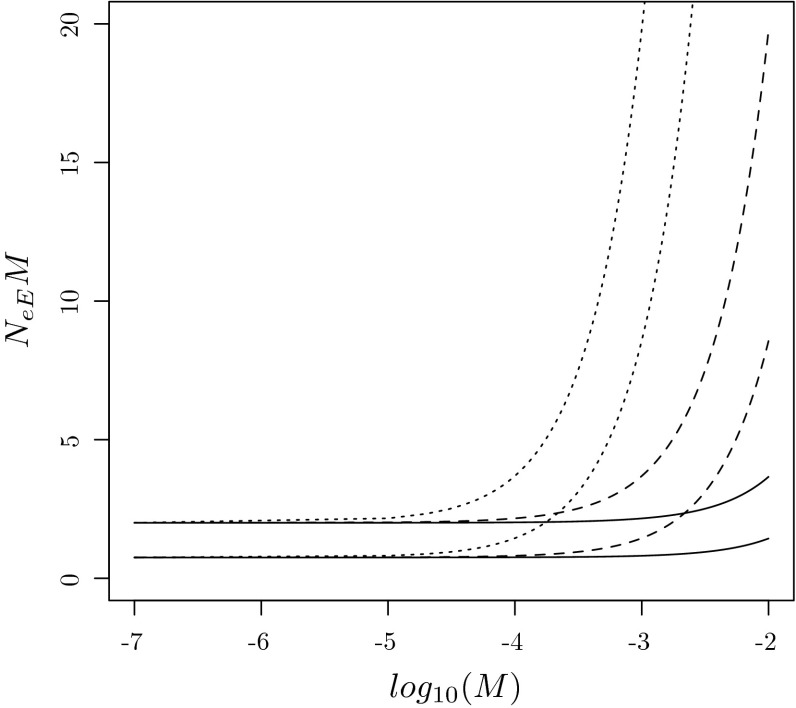
Plots of $N_{eE}M$ versus the migration rate $M$ for the island model, with $s=4$ (*lower curves*) and $s=9$ (*upper curves*), when the local census size $N/s$ equals 20 (*solid*), 200 (*dashed*) or 2,000 (*dotted*). The *upper curves* converge to $(9-1)/4=2$ and the *lower curves* to $(4-1)/4=3/4$ as $M\rightarrow 0$, in accordance with (125)

We finally apply (122) and arrive at

125 $$\begin{aligned} N_{eE}&= \frac{\frac{1}{s}\sum _{i=1}^s (-1)}{2(-\frac{2}{s-1})}\cdot \frac{1}{M} + o(M^{-1})\nonumber \\&= \frac{s-1}{4}\cdot \frac{1}{M} + o(M^{-1}) \end{aligned}$$

as $M\rightarrow 0$. The accuracy of this formula is illustrated in Fig. 1. $\square$

#### *Example 13*

*(Circular stepping stone model)*. The circular stepping stone model (Kimura 1953; Kimura and Weiss 1964; Maruyama 1970a) is a spatial model with $m=s$ demes located along the perimeter of a circle, where migration from any deme is only possible to one of its two nearest neighbors. The elements of the expected forward migration matrix are

126 $$\begin{aligned} M_{ki}(\varepsilon ) = \left\{ \begin{array}{ll} 1 - \varepsilon , &{} \text{ if } k=i,\\ \varepsilon /2, &{} \text{ if } \delta (k,i)=1,\\ 0, &{} \text{ otherwise }, \end{array}\right. \end{aligned}$$

where $\delta (i,j)$ is the shortest distance between demes $i$ and $j$ along the circle perimeter, when the distance between two neighboring demes is normalized to 1. It follows from (4), (7) and (8) that ${\varvec{M}}={\varvec{B}}$, $M=B=\varepsilon$ and $u_k(\varepsilon )=\gamma _k(\varepsilon )=1/s$. Hence

$$\begin{aligned} \dot{M}_{ki}=\dot{B}_{ik} = \left\{ \begin{array}{ll} -1, &{} k=i,\\ 1/2, &{} \delta (k,i)=1,\\ 0, &{} \text{ otherwise }, \end{array}\right. \end{aligned}$$

and from (120) we find that the matrix $\dot{{\varvec{\Lambda }}}=(\dot{\Lambda }_{ij,kl})$ has elements

$$\begin{aligned} \dot{\Lambda }_{ij,kl} = \left\{ \begin{array}{ll} -2, &{} k=i\ne j=l,\\ 1/2, &{} k=i\ne j, \delta (j,l)=1,\\ 1/2, &{} \delta (k,i)=1, i\ne j=l,\\ 0, &{} \text{ otherwise }. \end{array}\right. \end{aligned}$$

Since $\sum _{k=1}^s u_k(0)\sum _{i;i\ne k}\dot{M}_{ki} = 1$, we finally deduce from (122) that

127 $$\begin{aligned} N_{eE} = \frac{1}{-2\lambda _{ \text{ max }}(\dot{{\varvec{\Lambda }}})}\cdot \frac{1}{M} + o(M^{-1}) \text{ as } M\rightarrow 0. \end{aligned}$$

It seems difficult to obtain an explicit expression for the multiplicative constant in (127), although Maruyama (1970a) derived an approximation

128 $$\begin{aligned} N_{eE} \approx \frac{s^2}{2\pi ^2} \cdot \frac{1}{M} + o(M^{-1}) \end{aligned}$$

for even $s$. In Table 1 we compare (127) and (128) for different $s$ and find a very good agreement. $\square$

**Table 1:** Comparison between the multiplicative constants $N_{eE}=C/M + o(M^{-1})$ in (127), and the approximative multiplicative constant $N_{eE}\approx C^{ \text{ appr }}/M + o(M^{-1})$ in (128), for a circular stepping stone model with $s$ subpopulations with migration rate $M\rightarrow 0$

$s$	$C$	$C^{ \text{ appr }}$
2	0.2500	0.2026
3	0.5000	0.4559
4	0.8536	0.8106
5	1.3090	1.2665
6	1.8660	1.8238
7	2.5245	2.4824
8	3.2843	3.2423
9	4.1454	4.1035
10	5.1079	5.0661

#### *Example 14*

**Fig. 2:**
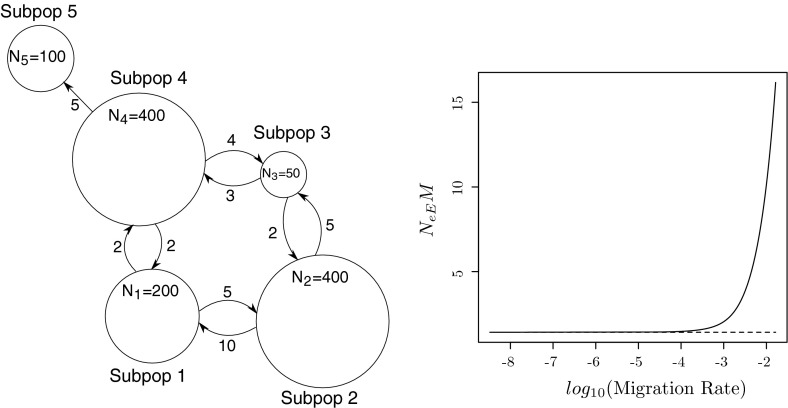
*Left* System with five subpopulations of sizes $N_i = Nu_i$ and, shown next to the *arrows*, the number of migrants $N_kM_{ki}$ between different pairs $k,i$ of subpopulations. *Right* Plots of $N_{eE}M$ versus the migration rate $M$ (*solid curve*) and the asymptotic limit $C=1.419$ (*dashed line*), calculated from the formula on the line below (131)

*(System with five subpopulations)*. A system with five subpopulations of varying size is shown in Fig. 2, with number of migrants in each generation depicted next to the arrows. The forward migration matrix is

$$\begin{aligned} {\varvec{M}}= \left( \begin{array}{l@{\quad }l@{\quad }l@{\quad }l@{\quad }l} 0.94 &{} 0.025 &{} 0 &{} 0.01 &{} 0\\ 0.025 &{} 0.9825 &{} 0.0125 &{} 0 &{} 0 \\ 0 &{} 0.04 &{} 0.82 &{} 0.06 &{} 0\\ 0.005 &{} 0 &{} 0.01 &{} 0.9875 &{} 0.0125\\ 0 &{} 0 &{} 0 &{} 0 &{} 0.95 \end{array}\right) , \end{aligned}$$

and the relative subpopulation size vector ${\varvec{u}}=(2,4,0.5,4,1)/11.5$. We let the forward migration rates depend on a perturbation parameter $\varepsilon$ according to

129 $$\begin{aligned} M_{ki}(\varepsilon ) = \left\{ \begin{array}{lll} \varepsilon M_{ki}, &{} i\ne k,\\ 1 + \varepsilon (M_{ii}-1), &{} i=k, \end{array}\right. \end{aligned}$$

so that ${\varvec{u}}={\varvec{u}}(\varepsilon )$ does not depend on $\varepsilon$, whereas the forward migration rate $M(\varepsilon )=\varepsilon M$ is proportional to $\varepsilon$. It follows from (105) that

130 $$\begin{aligned} \dot{B}_{ik} = \left\{ \begin{array}{lll} \frac{u_k}{u_i}M_{ki}, &{} k\ne i,\\ (M_{ii}-1), &{} k=i. \end{array}\right. \end{aligned}$$

Combining (122) and (129), we find that

131 $$\begin{aligned} N_{eE} = \frac{C}{M} + o(M^{-1}) \text{ as } M\rightarrow 0, \end{aligned}$$

with $C = (1-\sum _k u_k M_{kk})/(-2\lambda _{ \text{ max }}(\dot{{\varvec{\Lambda }}}))$ and $\dot{{\varvec{\Lambda }}}$ derived from (120) and (130). The numerically computed value $C=1.419$ is justified in Fig. 2. $\square$

#### *Example 15*

*(Combined spatial and age structure)*. Continuing Example 7, we assume that the forward migration matrix depends on $\varepsilon$ as

$$\begin{aligned} {\varvec{M}}(\varepsilon ) = {\varvec{M}}(0) + \varepsilon \dot{{\varvec{M}}}= \left( \begin{array}{ll} {\varvec{M}}_{11}(0)-\varepsilon \dot{{\varvec{M}}}_{12} &{} \varepsilon \dot{{\varvec{M}}}_{12} \\ \varepsilon \dot{{\varvec{M}}}_{21} &{} {\varvec{M}}_{22}(0)-\varepsilon \dot{{\varvec{M}}}_{21} \end{array}\right) , \end{aligned}$$

so that the two demes are isolated when $\varepsilon =0$. For brevity, write $M_{ki}=M_{ki}(0)$. The nonzero elements of the two off-diagonal blocks of $\dot{{\varvec{M}}}$ are

132 $$\begin{aligned} \begin{array}{llll} \dot{M}_{k,z+1} &{}=&{} c_1 M_{k1}u_{z+1}, &{} k=1,\ldots ,s,\\ \dot{M}_{z+k,1} &{}=&{} c_1 M_{z+k,z+1}u_1, &{} k=1,\ldots ,s,\\ \dot{M}_{k,z+k+1} &{}=&{} c_{k+1}M_{k,k+1}u_{z+k+1}, &{} k=1,\ldots ,s-1,\\ \dot{M}_{z+k,k+1} &{}=&{} c_{k+1}M_{z+k,z+k+1}u_{k+1}, &{} k=1,\ldots ,s-1, \end{array} \end{aligned}$$

where $c_1,\ldots ,c_z$ are non-negative constants, of which at least one is strictly positive. The migration rates in (132) are chosen so that $u_k=u_k(\varepsilon )$ does not depend on $\varepsilon$. Intuitively, a fraction $\varepsilon c_1 u_{z+1}$ of all offspring in deme 1 end up in deme 2, and a fraction $\varepsilon c_{k+1}u_{z+k+1}$ of all genes of age class $k$ of deme 1 that survive, migrate to deme 2, and similarly for the other two equations of (132). In the “Appendix” we verify that

133 $$\begin{aligned} N_{eE} = \frac{\sum _{i=1}^z c_i u_iu_{z+i}}{\sum _{i=1}^z c_i(\gamma _{1i}u_{z+i}+\gamma _{2i}u_i)}\cdot \frac{1}{M} + o(M^{-1}) \end{aligned}$$

as $M\rightarrow 0$. When $c_i$ increases with $i$, older individuals will migrate more, and this will increase $N_{eE}$ if older individuals are less reproductive, and decrease $N_{eE}$ if they reproduce more. Conservative migration is the intermediate case when all age groups are equally reproductive, with $\gamma _{1i}=u_i/U(1)$, $\gamma _{2i}=u_{z+i}/U(2)$ and $U(d)=\sum _{i\in \mathcal{I}(d)} u_i$ the relative size of deme $d$. Insertion into (133) gives

$$\begin{aligned} N_{eE} = \frac{U(1)U(2)}{M} + o(M^{-1}) \end{aligned}$$

as $M\rightarrow 0$ for conservative migration, independently of the age dependency of the migration pattern. In particular, if both demes are equally large, we get the same multiplicative constant $C=U(1)U(2)=(1/2)^2 = 1/4$ as for an island model (125) with $s=2$. $\square$

### Large populations and small migration rates

We let the inverse population size and the backward migration rate both tend to zero at the same speed, so that

134 $$\begin{aligned} {\varvec{B}}(\varepsilon )&= {\varvec{B}}(0) + \varepsilon \dot{{\varvec{B}}},\nonumber \\ 4N\varepsilon&= c, \end{aligned}$$

with $\varepsilon \rightarrow 0$ and $c$ a constant. This can be viewed as an asymptotic scenario intermediate between (74) (with $\beta =1$) and (102).

The asymptotic expression for $N_{eE}$ is derived similarly as in the previous subsection, so we only highlight the differences. Since the population size tends to infinity, the coalescence probabilities $p_{ijk}$ will tend to zero, as described in (77), and this modifies (73) to

135 $$\begin{aligned} A_{ij,kl}(\varepsilon ) = \left( 1-\frac{2\varepsilon }{cu_i}\right) ^{\{i=j\}} \left( \frac{1-\frac{2\sigma _{ijk}\varepsilon }{cu_k}}{1-\frac{2\varepsilon }{cu_k}} \right) ^{\{k=l\}} Q_{ij,kl}(\varepsilon ) + o(\varepsilon ), \end{aligned}$$

where $\sigma _{ijk}$ is the size standardized coalescence rate in (77). Consequently,

$$\begin{aligned} {\varvec{A}}(0) = {\varvec{Q}}(0) = \text{ diag }\left( {\varvec{A}}_{ab}(0); 1\le a,b \le m\right) \end{aligned}$$

is a block diagonal matrix with blocks ${\varvec{A}}_{ab}(0)$ given by (115) when $\varepsilon =0$ for *all* $1\le a,b \le m$. These blocks have a unique largest eigenvalue 1, and

$$\begin{aligned} v = |\{\alpha =(a,b); \, 1\le a,b \le m\}| = m^2. \end{aligned}$$

When $a\ne b$, the left and right eigenvectors of ${\varvec{A}}_{ab}(0)$ are as in (116). The same is true when $a=b$ if we add the assumption of fixed or multinomial backward migration proportions. Differentiating (135) with respect to $\varepsilon$ we find that

136 $$\begin{aligned} \dot{A}_{ij,kl} = \dot{Q}_{ij,kl} - \frac{2}{c}\left( 1_{\{k= l\}} \frac{\sigma _{ijk}-1}{u_k} +\frac{1_{\{i=j\}}}{u_i}\right) Q_{ij,kl}(0), \end{aligned}$$

with $\dot{Q}_{ij,kl}$ as in (117), but without the restriction $a\ne b$. Therefore, inserting (116) and (136) into the definition of $\dot{{\varvec{\Lambda }}}= (\dot{\Lambda }_{ab,cd})_{1\le a,b\le m, 1\le c,d \le m}$ in (71), we find, after some computations, that this matrix has elements

137 $$\begin{aligned} \dot{\Lambda }_{ab,cd} = \left\{ \begin{array}{l@{\quad }l} G_{aa}+G_{bb} - 1_{\{a=b\}}2C_a/c, &{} c=a,d=b,\\ G_{bd}, &{} c=a, d \ne b,\\ G_{ac}, &{} c\ne a, d=b,\\ 0, &{} c\ne a, d \ne b, \end{array}\right. \end{aligned}$$

with $G_{ab}$ as in (108) and

$$\begin{aligned} C_a = \sum _{i,j,k\in \mathcal{I}(a)} \frac{\gamma _{ai}\gamma _{aj} \sigma _{ijk} B_{ik}(0)B_{jk}(0)}{u_k} \end{aligned}$$

is a coalescence rate between the lines of deme $a$ that can be interpreted as a local version of (90). In particular, when each subpopulation $i$ is a deme, $G_{ik}=\dot{B}_{ik}$, and (137) reduces to

138 $$\begin{aligned} \dot{\Lambda }_{ij,kl} = \left\{ \begin{array}{l@{\quad }l} \dot{B}_{ii}+\dot{B}_{jj} - 1_{\{i=j\}}2\sigma _{iii}/(cu_i), &{} k=i,l=j,\\ \dot{B}_{jl}, &{} k=i, l \ne j,\\ \dot{B}_{ik}, &{} k\ne i, l=j,\\ 0, &{} k\ne i, l \ne j. \end{array}\right. \end{aligned}$$

Equipped with (137) and (138), we apply (69) and Theorem 4, and deduce:

#### **Proposition 4**

Suppose the migration rate between demes and the inverse population size tend to zero simultaneously as in (134) when $\varepsilon \rightarrow 0$, with a backward migration that is either fixed (44) or multinomial (48). The eigenvalue effective size then has an asymptotic expansion

139 $$\begin{aligned} N_{eE} = - \frac{1}{2\lambda _{ \text{ max }}(\dot{{\varvec{\Lambda }}})\varepsilon } + o(\varepsilon ^{-1}), \end{aligned}$$

with the elements of $\dot{{\varvec{\Lambda }}}$ as in (137), or (138) when each deme contains one single subpopulation.

#### *Example 16*

*(Island model.)* We will assume that

140 $$\begin{aligned} \sigma _{iii}=\sigma \text{ for } i=1,\ldots ,s, \end{aligned}$$

where $\sigma =(N/s)/N_{eI}$ can be interpreted as a ratio between the (constant) local census and effective size of each deme. This local inbreeding effective size $N_{eI}=N_{eI,i}$ is similar to (53), although we only consider triplets $i=j=k$ of demes here. It follows from (124), (138) and (140) that

141 $$\begin{aligned} \dot{\Lambda }_{ij,kl} = \left\{ \begin{array}{l@{\quad }l} -2 - 1_{\{i=j\}}2s\sigma /c, &{} k=i,l=j,\\ \frac{1}{s-1}, &{} k=i, l \ne j,\\ \frac{1}{s-1}, &{} k\ne i, l=j,\\ 0, &{} k\ne i, l \ne j. \end{array}\right. \end{aligned}$$

**Fig. 3:**
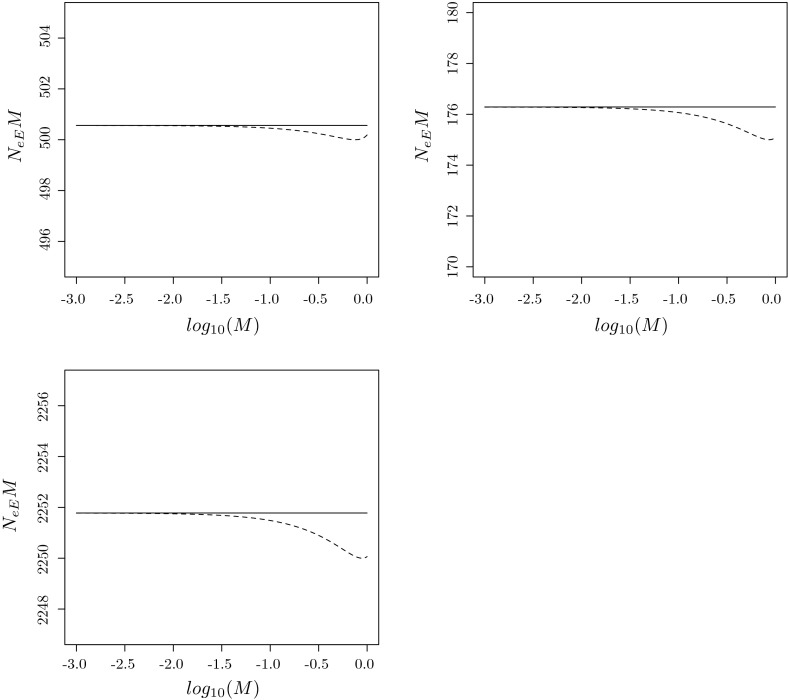
Plots of $N_{eE}M$ versus $M$ for the island model, when $N_{el}=N/s$ and $\kappa =4(N/s)M$ is kept constant, with $s=4$ and $\kappa =500$ (*upper left*), $s=7$ and $\kappa =100$ (*upper right*) and $s=9$ and $\kappa =1,000$ (*lower left*). The *horisontal lines* are the limits $1/(-2\dot{\lambda }(\kappa ))$ of $N_{eE}M$ in (143) as $M\rightarrow 0$

Let $\dot{\lambda }= \lambda _{ \text{ max }}(\dot{{\varvec{\Lambda }}})$ be the largest eigenvalue of $\dot{{\varvec{\Lambda }}}$, with ${\varvec{x}}= \text{ vec }\left( (x_{ij})_{i,j=1}^s\right)$ the corresponding right eigenvector satisfying $\dot{{\varvec{\Lambda }}}{\varvec{x}}= \dot{\lambda }{\varvec{x}}$. By symmetry we must have $x_{ij} = y$ when $i=j$ and $x_{ij}=z$ when $i\ne j$, and a $2\times 2$ system

142 $$\begin{aligned} \left\{ \begin{array}{rcl} \dot{\lambda }y &{}=&{} (\dot{{\varvec{\Lambda }}}{\varvec{x}})_{ii} = 2(z-y) - \frac{2s\sigma }{c}y\\ \dot{\lambda }z &{}\mathop {=}\limits ^{i\ne j}&{} (\dot{{\varvec{\Lambda }}}{\varvec{x}})_{ij} = \frac{2}{s-1}(y-z) \end{array}\right. \end{aligned}$$

of equations for $y$ and $z$. It will be convenient to introduce the parameter $\kappa = c/(s\sigma ) = 4NM/(s\sigma ) =: 4N_{eI}M$. Then we apply (139) and find that

143 $$\begin{aligned} N_{eE} = \frac{1}{-2\dot{\lambda }(\kappa ) M} + o(M^{-1}) \end{aligned}$$

as $$M\rightarrow 0$$, where

$$\begin{aligned} \dot{\lambda }(\kappa ) = - \left( \frac{s}{s-1}+ \frac{1}{\kappa }\right) \left( 1-\sqrt{1- \frac{4\kappa }{(s-1)\left( \frac{s\kappa }{s-1}+ 1\right) ^2}}\right) \end{aligned}$$

is the largest root of the (quadratic) characteristic equation in $\dot{\lambda }$ obtained from (142). Figure 3 verifies numerically fast convergence in (143), for three combinations of $s$ and $\kappa$ (notice the narrow scales of the $y$-axes). We have that $\lim _{\kappa \rightarrow 0} (-2\dot{\lambda }(\kappa )) = 4/(s-1)$, in agreement with (125). On the other hand,

$$\begin{aligned} N_{eE} = \frac{N}{C(\kappa )} + o(N^{-1}), \end{aligned}$$

where

$$\begin{aligned} C(\kappa ) = \sigma \cdot \left( \frac{s^2\kappa }{2(s-1)} +\frac{s}{2}\right) \left( 1-\sqrt{1-\frac{4\kappa }{(s-1) \left( \frac{s\kappa }{s-1}+1\right) ^2}}\right) , \end{aligned}$$

and when the migration rate dominates the inverse population size, we get $\lim _{\kappa \rightarrow \infty } C(\kappa ) = \sigma$ for $\sigma _k=\sigma$ and $\gamma _k=u_k=1/s$, in agreement with (96). $\square$

## Discussion

In this paper we developed a general theory which enables computation of the eigenvalue effective size $N_{eE}$ for a large class of structured populations with stochastic backward migration and exchangeable reproduction within subpopulations, exactly or asymptotically when either the inverse population size and/or migration rates between subpopulations tend to zero.

Our work can be extended in several ways. First, subpopulation sizes could be time varying. Existence of $\lambda$ then requires extra conditions, e.g. sizes that either vary as a Markov chain or cyclically. Several authors have studied this problem for homogeneous or age structured models, see Karlin (1968), Jagers and Sagitov (2004), Pollak (1980, 2002) and Wang and Pollak (2000a, b). For cyclically varying populations with period $\tau$, the matrix ${\varvec{A}}={\varvec{A}}_t$ of the predicted gene diversity recursion will depend cyclically on time. Whitlock and Barton (1997) argued that this deterministic process tends to zero at a rate

144 $$\begin{aligned} \lambda = \lambda _{ \text{ max }}\left( {\varvec{A}}_\tau \cdot \ldots \cdot {\varvec{A}}_1\right) ^{1/\tau }, \end{aligned}$$

as formally proved in Hössjer et al. (2014). It is straightforward to extend Theorem 1 by measuring time in units of $\tau$, so that the allele frequency Markov process has kernel ${\varvec{P}}_1\cdot \ldots \cdot {\varvec{P}}_\tau$. Then (144) equals the rate of fixation $\lambda = \lambda _{3}\left( {\varvec{P}}_1\cdot \ldots \cdot {\varvec{P}}_\tau \right) ^{1/\tau }$ of alleles in units of time step one.

Second, we have included two-sex models, defining subpopulations in terms of male and female gametes. It would also be of interest to define subpopulations in terms of individuals, as for an island model with diploid monoecious or dioecious individuals (Chesser et al. 1993; Wang 1997a, b). This would require some changes in the way the elements of ${\varvec{A}}$ are characterized in terms of coalescence probabilities, requiring modifications of (34) and Theorem 3.

## Appendix

### *Proof of Theorem 1*

Formula (14) follows from the lower block diagonal decomposition (13) of ${\varvec{P}}$, which implies that the spectrum of eigenvalues of ${\varvec{P}}$ is the union of the spectrum of eigenvalues of all ${\varvec{P}}_{ii}$. Moreover, for each $i\ge 3$ we have that $\lambda _{ \text{ max }}({\varvec{P}}_{ii}) <1$, since ${\varvec{P}}_{ii}$ has non-negative elements, row sums less or equal to one, with at least one row sum strictly less than one. In conjunction with (14), this implies $\lambda _3<1$.

In order to prove (15), we recall from (12) that $\phi _2({\varvec{x}})=\gamma {\varvec{x}}$. It follows from (10) that $\{\phi _2({\varvec{X}}_t)\}$ is a martingale. With $\tau = \min \{t; \, {\varvec{X}}_t\in \mathcal{X}_1\cup \mathcal{X}_2\}$ a stopping time, we can use the Optional Stopping Theorem (cf. Chapter 7 of Grimmett and Stirzaker 2001) to deduce

145 $$\begin{aligned} \lim _{t\rightarrow \infty } {\varvec{P}}^{t}({\varvec{x}},{\varvec{1}})&= P_{{\varvec{x}}}({\varvec{X}}_\tau = {\varvec{1}})\nonumber \\&= E_{{\varvec{x}}}(\phi _2({\varvec{X}}_\tau ))\nonumber \\&= E_{{\varvec{x}}}(\phi _2({\varvec{X}}_0))\nonumber \\&= \phi _2({\varvec{x}}), \end{aligned}$$

and similarly ${\varvec{P}}^{t}({\varvec{x}},{\varvec{0}}) \rightarrow 1-\phi _2({\varvec{x}}) = \phi _1({\varvec{x}})$, for any ${\varvec{x}}\in \mathcal{X}$. This establishes the leading two terms on the right hand side of (15). In order motivate the next term of order $\lambda _3^t$, we notice that ${\varvec{P}}_{kk}$ is non-negative, irreducible and aperiodic, and therefore has a unique largest eigenvalue $\lambda _3$. But since the maximum in (14) is attained uniquely for $i=k$, $\lambda _3$ must also be a simple eigenvalue for ${\varvec{P}}$, so that $|\lambda _i|<\lambda _3$ for $i=4,\ldots ,|\mathcal{X}|$. In conjunction with (145), this implies

146 $$\begin{aligned} {\varvec{P}}^t = {\varvec{\phi }}_1{\varvec{\pi }}_1 + {\varvec{\phi }}_2{\varvec{\pi }}_2 + \lambda ^t {\varvec{R}}+ o(\lambda ^t), \end{aligned}$$

for some matrix ${\varvec{R}}$ as $t\rightarrow \infty$. Since ${\varvec{P}}$ has a lower triangular block decomposition by (11), so has ${\varvec{P}}^t=({\varvec{P}}^{(t)}_{ij})$ and ${\varvec{R}}=({\varvec{R}}_{ij})$. Moreover, ${\varvec{P}}^t$ has non-negative elements and ${\varvec{\phi }}_1{\varvec{\pi }}_1 + {\varvec{\phi }}_2{\varvec{\pi }}_2$ has all but its first two columns equal to zero, and therefore each ${\varvec{R}}_{ij}$ with $j\ge 3$ is non-negative. Apply Perron-Frobenius Theorem to the non-negative, irreducible and aperiodic matrix ${\varvec{P}}_{kk}$ in order to deduce ${\varvec{P}}_{kk}^{(t)} = \lambda _3^t{\varvec{\phi }}_k{\varvec{q}}_k + o(\lambda _3^t)$, with ${\varvec{\phi }}_k$ and ${\varvec{q}}_k$ the right and left eigenvectors for the leading eigenvalue $\lambda _3$ with strictly positive components, normalized for instance so that $\sum _{{\varvec{x}}\in \mathcal{X}_k} q_k({\varvec{x}}) = \sum _{{\varvec{x}}\in \mathcal{X}_k} \phi _k({\varvec{x}})q_k({\varvec{x}}) = 1$. This proves that ${\varvec{R}}_k = {\varvec{\phi }}_k{\varvec{q}}_k$, and since all other block diagonal ${\varvec{P}}_{ii}$ have leading eigenvalues smaller than $\lambda _3$, the corresponding matrices ${\varvec{R}}_{ii}$ must be zero.

The remaining submatrices ${\varvec{R}}_{ij}$ of ${\varvec{R}}$ ($i>j\ge 1$, $i\ge 3$) can be computed as follows. We use (15) and the two recursions ${\varvec{P}}^{(t+1)}_{ij} = \sum _{l=j}^i {\varvec{P}}_{il}{\varvec{P}}_{lj}^{(t)}$ and ${\varvec{P}}^{(t+1)}_{ij} = \sum _{l=j}^i {\varvec{P}}_{il}^{(t)}{\varvec{P}}_{lj}$ and let $t\rightarrow \infty$. After some computations this leads to

147 $$\begin{aligned} \begin{array}{rcll} {\varvec{R}}_{ij} &{}=&{} (\lambda _3{\varvec{I}}_{|\mathcal{X}_i|}-{\varvec{P}}_{ii})^{-1}\sum _{l=\max (j,3)}^{i-1} {\varvec{P}}_{il}{\varvec{R}}_{lj}, &{} i=j+1,\ldots ,n,\\ {\varvec{R}}_{ij} &{}=&{} \sum _{l=\max (j+1,3)}^i {\varvec{R}}_{il}{\varvec{P}}_{lj}(\lambda _3 {\varvec{I}}_{|\mathcal{X}_j|}-{\varvec{P}}_{jj})^{-1}, &{} j=i-1,\ldots ,1, \end{array} \end{aligned}$$

with ${\varvec{I}}_p$ an identity matrix of order $p$. The upper and lower recursions of (147) is applicable for $i\ne k$ and $j\ne k$, and all ${\varvec{R}}_{ij}$ can be found from them. $\square$

### *Proof of Corollary 1*

It follows from (15) and (17) that

$$\begin{aligned} E_\pi (\phi (X_t))&= {\varvec{\pi }} {\varvec{P}}^t {\varvec{\phi }}\\&= ({\varvec{\pi }}{\varvec{\phi }}_1)({\varvec{\pi }}_1{\varvec{\phi }}) + ({\varvec{\pi }}{\varvec{\phi }}_2)({\varvec{\pi }}_2{\varvec{\phi }}) + \lambda _3^t{\varvec{\pi }} {\varvec{R}}{\varvec{\phi }} + o(\lambda _3^t)\\&= \lambda _3^t \sum _{{\varvec{x}},{\varvec{y}}\in \mathcal{X}} \pi ({\varvec{x}}){\varvec{R}}({\varvec{x}},{\varvec{y}})\phi ({\varvec{y}}) + o(\lambda _3^t)\\&= \lambda _3^t \sum _{{\varvec{x}}\in \mathcal{X},{\varvec{y}}\in \mathcal{X}\setminus (\mathcal{X}_1\cup \mathcal{X}_2)} \pi ({\varvec{x}}){\varvec{R}}({\varvec{x}},{\varvec{y}})\phi ({\varvec{y}}) + o(\lambda _3^t)\\&\ge \lambda _3^t \sum _{{\varvec{x}},{\varvec{y}}\in \mathcal{X}_k} \pi ({\varvec{x}}){\varvec{R}}({\varvec{x}},{\varvec{y}})\phi ({\varvec{y}}) + o(\lambda _3^t)\\&= \lambda _3^t \sum _{{\varvec{x}},{\varvec{y}}\in \mathcal{X}_k} \pi ({\varvec{x}})\phi _k({\varvec{x}})q_k({\varvec{y}})\phi ({\varvec{y}}) + o(\lambda _3^t)\\&= \lambda _3^t \sum _{{\varvec{x}}\in \mathcal{X}_k} \pi ({\varvec{x}})\phi _k({\varvec{x}}) \sum _{{\varvec{y}}\in \mathcal{X}_k} q_k({\varvec{y}})\phi ({\varvec{y}}) + o(\lambda _3^t) \\&> 0, \end{aligned}$$

where the last inequality holds if $\pi (\mathcal{X}_k)>0$, since $q_k$, $\phi _k$ and $\phi$ are strictly positive functions on $\mathcal{X}_k$. $\square$

### *Proof of Proposition 2*

The proposition follows from

$$\begin{aligned} E\left( \phi _{{\varvec{W}}}({\varvec{X}}_t)\right)&= 2\sum _{i,j=1}^s W_{ij}E_\pi \left( X_{ti}(1-X_{tj})\right) \nonumber \\&= \sum _{i,j=1}^s W_{ij}\left( E_\pi \left( X_{ti}(1-X_{tj})\right) + E_\pi \left( X_{tj}(1-X_{ti})\right) \right) \nonumber \\&= \sum _{i,j=1}^s W_{ij}H_{tij}, \end{aligned}$$

where in the second step we used the symmetry condition $W_{ij}=W_{ji}$, and in the last step the definition of $H_{tij}$ in (23). $\square$

### *Proof of Theorem 2*

Assume that all elements $W_{ij}$ of ${\varvec{W}}$ are strictly positive, and that the symmetry condition $W_{ji}=W_{ij}$ holds. Then $\phi _{{\varvec{W}}}$ satisfies (17), and it follows from (18) and Proposition 2 that

148 $$\begin{aligned} {\varvec{W}}{\varvec{H}}_t = C \lambda ^t + o(\lambda ^t) \text{ as } t\rightarrow \infty , \end{aligned}$$

with $C > 0$. On the other hand we can use (25) repeatedly $t$ times to deduce

149 $$\begin{aligned} {\varvec{W}}{\varvec{H}}_t = {\varvec{W}}{\varvec{A}}^t {\varvec{H}}_0. \end{aligned}$$

The block decomposition (28) implies that $\lambda _{ \text{ max }}({\varvec{A}}) = \max _{a=1,\ldots ,m} \lambda _{ \text{ max }}({\varvec{A}}_{aa})$, and since (148)–(149) hold for any vector ${\varvec{W}}$ with non-negative components and any admissible ${\varvec{H}}_0$, (29) follows. In the sequel, we therefore write $\lambda =\lambda _{ \text{ max }}({\varvec{A}})$.

If the maximum in (29) is attained for a unique $1\le c\le m$, we lump $\mathcal{I}_2 = \bar{\mathcal{C}}_1 \cup \bar{\mathcal{C}}_2 \cup \bar{\mathcal{C}}_3$ into (at most) three components $\bar{\mathcal{C}}_1 = \cup _{a=1}^{c-1} \mathcal{C}_a$, $\bar{\mathcal{C}}_2 = \mathcal{C}_c$ and $\bar{\mathcal{C}}_3 = \cup _{a=c+1}^{m} \mathcal{C}_a$. In particular, we put $\bar{\mathcal{C}}_1=\emptyset$ if $c=1$, and $\bar{\mathcal{C}}_3=\emptyset$ if $c=m$. The corresponding block decomposition of ${\varvec{A}}$ is

150 $$\begin{aligned} {\varvec{A}}= \left( \begin{array}{c@{\quad }c@{\quad }c} \bar{{\varvec{A}}}_{11} &{} {\varvec{0}}&{} {\varvec{0}}\\ \bar{{\varvec{A}}}_{21} &{} \bar{{\varvec{A}}}_{22} &{} {\varvec{0}}\\ \bar{{\varvec{A}}}_{31} &{} \bar{{\varvec{A}}}_{32} &{} \bar{{\varvec{A}}}_{33} \end{array}\right) . \end{aligned}$$

It can be shown that matrices ${\varvec{C}}_{ab}$ exist for $1\le b \le a \le 3$ such that

151 $$\begin{aligned} {\varvec{A}}^t = \left( \begin{array}{c@{\quad }c@{\quad }c} \bar{{\varvec{A}}}_{11}^{(t)} &{} {\varvec{0}}&{} {\varvec{0}}\\ \bar{{\varvec{A}}}_{21}^{(t)} &{} \bar{{\varvec{A}}}_{22}^{(t)} &{} {\varvec{0}}\\ \bar{{\varvec{A}}}_{31}^{(t)} &{} \bar{{\varvec{A}}}_{32}^{(t)} &{} \bar{{\varvec{A}}}_{33}^{(t)} \end{array}\right) = \lambda ^t\left( \begin{array}{c@{\quad }c@{\quad }c} {\varvec{C}}_{11} &{} {\varvec{0}}&{} {\varvec{0}}\\ {\varvec{C}}_{21} &{} {\varvec{C}}_{22} &{} {\varvec{0}}\\ {\varvec{C}}_{31} &{} {\varvec{C}}_{32} &{} {\varvec{C}}_{33} \end{array}\right) + o(\lambda ^t) \end{aligned}$$

as $t\rightarrow \infty$. We will identify the components of ${\varvec{C}}=({\varvec{C}}_{ab})$, and in this process find the right and left eigenvectors ${\varvec{r}}$ and ${\varvec{\rho }}$ of ${\varvec{A}}$.

Starting with the diagonal submatrices of ${\varvec{C}}$, it follows that ${\varvec{C}}_{11}={\varvec{C}}_{33}={\varvec{0}}$, since $\lambda _{ \text{ max }}(\bar{{\varvec{A}}}_{aa}) < \lambda$ for $a=1,3$. On the other hand, since $\bar{{\varvec{A}}}_{22}={\varvec{A}}_{cc}$ is irreducible with $\lambda _{ \text{ max }}(\bar{{\varvec{A}}}_{aa}) = \lambda$, Perron–Frobenius Theorem and (149) imply that $\lambda$ is a simple eigenvalue of $\bar{{\varvec{A}}}_{22}$ with periodicity $1$. Thus we can find a right eigenvector ${\varvec{r}}_2 = \left( r_{2ij};\, ij\in \bar{\mathcal{C}}_2\right) ^\prime$, and a left eigenvector ${\varvec{\rho }}_2 = \left( \rho _{2ij};\, ij\in \bar{\mathcal{C}}_2\right)$ of $\bar{{\varvec{A}}}_{22}$ with strictly positive components, normalized so that $\sum _{ij\in \bar{\mathcal{C}}_2} \rho _{2ij} = \sum _{ij\in \bar{\mathcal{C}}_2} r_{2ij}\rho _{2ij} = 1$, with

152 $$\begin{aligned} {\varvec{C}}_{22} = {\varvec{r}}_2{\varvec{\rho }}_2. \end{aligned}$$

For the non-diagonal elements of ${\varvec{C}}$ we use the three recursions

153 $$\begin{aligned} \bar{{\varvec{A}}}^{(t+1)}_{21}&= \bar{{\varvec{A}}}^{(t)}_{21}\bar{{\varvec{A}}}_{11} + \bar{{\varvec{A}}}^{(t)}_{22} \bar{{\varvec{A}}}_{21},\nonumber \\ \bar{{\varvec{A}}}^{(t+1)}_{32}&= \bar{{\varvec{A}}}_{32}\bar{{\varvec{A}}}^{(t)}_{22} + \bar{{\varvec{A}}}_{33} \bar{{\varvec{A}}}^{(t)}_{32},\nonumber \\ \bar{{\varvec{A}}}^{(t+1)}_{31}&= \bar{{\varvec{A}}}_{32}\bar{{\varvec{A}}}^{(t)}_{21} + \bar{{\varvec{A}}}_{33} \bar{{\varvec{A}}}^{(t)}_{31} + o(\lambda ^t), \end{aligned}$$

insert (151) into (153), divide both sides of all three equations in (153) by $\lambda ^t$ and let $t\rightarrow \infty$. After some computations, this yields

154 $$\begin{aligned} {\varvec{C}}_{21}&= {\varvec{C}}_{22}\bar{{\varvec{A}}}_{21}(\lambda {\varvec{I}}_{|\bar{\mathcal{C}}_1|}-\bar{{\varvec{A}}}_{11})^{-1},\nonumber \\ {\varvec{C}}_{32}&= (\lambda {\varvec{I}}_{|\bar{\mathcal{C}}_3|}-\bar{{\varvec{A}}}_{33})^{-1} \bar{{\varvec{A}}}_{32}{\varvec{C}}_{22},\nonumber \\ {\varvec{C}}_{31}&= (\lambda {\varvec{I}}_{|\bar{\mathcal{C}}_3|}-\bar{{\varvec{A}}}_{33})^{-1} \bar{{\varvec{A}}}_{32}{\varvec{C}}_{22}\bar{{\varvec{A}}}_{21}(\lambda {\varvec{I}}_{|\bar{\mathcal{C}}_1|}-\bar{{\varvec{A}}}_{11})^{-1}, \end{aligned}$$

with ${\varvec{I}}_p$ an identity matrix of order $p$. Finally, inserting (152) into (154), we find that ${\varvec{C}}= {\varvec{r}}{\varvec{\rho }} = ({\varvec{0}}^\prime ,{\varvec{r}}_2^\prime ,{\varvec{r}}_3^\prime )^\prime ({\varvec{\rho }}_1,{\varvec{\rho }}_2,{\varvec{0}})$, with

155 $$\begin{aligned} {\varvec{\rho }}_1&= {\varvec{\rho }}_2\bar{{\varvec{A}}}_{21}(\lambda {\varvec{I}}_{|\mathcal{C}_1|}- \bar{{\varvec{A}}}_{11})^{-1},\nonumber \\ {\varvec{r}}_3&= (\lambda {\varvec{I}}_{|\mathcal{C}_3|}-\bar{{\varvec{A}}}_{33})^{-1} \bar{{\varvec{A}}}_{32} {\varvec{r}}_2. \end{aligned}$$

Both ${\varvec{r}}_3$ and ${\varvec{\rho }}_1$ must have non-negative elements, since ${\varvec{r}}_2$ and ${\varvec{\rho }}_2$ have strictly positive elements, all $\bar{{\varvec{A}}}_{ab}$ are non-negative and $\bar{{\varvec{A}}}_{11},\bar{{\varvec{A}}}_{33}$ have all their eigenvalues less than $\lambda$. Finally, since ${\varvec{r}}_2$ and ${\varvec{\rho }}_2$ are right and left eigenvectors of $\bar{{\varvec{A}}}_{22}$ with eigenvalue $\lambda$, it follows after some computations from (155) and the block decomposition (150) of ${\varvec{A}}$, that ${\varvec{r}}$ and ${\varvec{\rho }}$ are right and left eigenvectors of ${\varvec{A}}$ with eigenvalue $\lambda$. $\square$

### *Proof of Theorem 3*

Recall that $\mathcal{H}_{tij}$ in (22) is the probability that two genes at time $t$ have different types of alleles when picked independently from subpopulations $i$ and $j$, with replacement if $i=j$. Conditionally on $\varvec{\mathcal {B}}_t$ and ${\varvec{X}}_{t-1}$, we compute the expected value of this probability by conditioning on the parental subpopulations $k$ and $l$ of $i$ and $j$, and then take into account whether the two parental genes are identical or not. We find that

156 $$\begin{aligned} E(\mathcal{H}_{tij}|\varvec{\mathcal {B}}_t,{\varvec{X}}_{t-1})=\left( 1-\frac{1}{2Nu_i}\right) ^{\{i =j\}} \sum _{k,l} \mathcal{Q}_{tij,kl}\mathcal{H}_{t-1,kl}\left( \frac{1-\mathcal{P}_{tijk}}{1-\frac{1}{2Nu_k}}\right) ^{\{k=l\}},\nonumber \\ \end{aligned}$$

since the probability of picking two different genes at time $t$ is $1-1/(2Nu_i)$ when $i=j$ and 1 when $i\ne j$. Then the probability that the two parental genes from subpopulations $k$ and $l$ are different is 1 when $k\ne l$ and $1-\mathcal{P}_{tijk}$ when $k=l$. In the former $k\ne l$ case, the probability is $\mathcal{H}_{t-1,kl}$ that the parental genes are different by state, and when $k=l$, the probability is $\mathcal{H}_{t-1,kk}/(1-(2Nu_k)^{-1})$ that the two parental genes are different by state.

We can express the coalescence probability $p_{ijk}$ in (37) in terms of $\mathcal{P}_{tijk}$ as

157 $$\begin{aligned} p_{ijk}&= P(T=1|I_0=i,J_0=j,I_1=J_1 = k,T>0)\nonumber \\&= P(T=1,I_1=J_1= k|I_0=i,J_0=j,T>0)\nonumber \\&/P(I_1=J_1 = k|I_0=i,J_0=j,T>0)\nonumber \\&= E\left( P(T=1,I_1=J_1= k|I_0=i,J_0=j,T>0,\varvec{\mathcal {B}}_t)\right) /Q_{ij,kk}\nonumber \\&= E\left( P(T=1|I_1=J_1 = k,I_0=i,J_0=j,T>0,\varvec{\mathcal {B}}_t)\right. \nonumber \\&\cdot \left. P(I_1=J_1 = k|I_0=i,J_0=j,T>0,\varvec{\mathcal {B}}_t)\right) /Q_{ij,kk}\nonumber \\&= E\left( \mathcal{P}_{tijk}\mathcal{Q}_{tij,kk}\right) /Q_{ij,kk}. \end{aligned}$$

Then average with respect to $\varvec{\mathcal {B}}_t$ on the left and right hand sides of (156), use independence between $\varvec{\mathcal {B}}_t$ and ${\varvec{X}}_{t-1}$, invoke (35), (41) and (157), to find that

158 $$\begin{aligned} E(\mathcal{H}_{tij}|{\varvec{X}}_{t-1})&= \left( 1-\frac{1}{2Nu_i}\right) ^{\{i=j\}} \sum _{k,l} Q_{ij,kl} \mathcal{H}_{t-1,kl} \left( \frac{1-p_{ijk}}{1- \frac{1}{2Nu_k}}\right) ^{\{k=l\}}\nonumber \\&= \sum _{k,l} A_{ij,kl} \mathcal{H}_{t-1,kl}, \end{aligned}$$

in accordance with (40). As a next step we average both sides of (158) with respect to ${\varvec{X}}_{t-1}$, using starting distribution $\pi$ for ${\varvec{X}}_0$, and get

159 $$\begin{aligned} H_{tij}&= E_\pi \left( E(\mathcal{H}_{tij}|{\varvec{X}}_{t-1})\right) \nonumber \\&= \sum _{k,l} A_{ij,kl} E_\pi (\mathcal{H}_{t-1,kl})\nonumber \\&= \sum _{k,l} A_{ij,kl} H_{t-1,kl}, \end{aligned}$$

which is equivalent to (25). To verify (39), we first compute

160 $$\begin{aligned}&P\left( T=1|I_0=i,J_0=j,I_1=J_1 =k,T>0,\varvec{\mathcal {B}}_t,\{{\varvec{\nu }}_{tkg}\}_{g =1}^{2Nu_k}\right) \nonumber \\&\quad = \sum _{g=1}^{2Nu_k} \nu _{tkig}(\nu _{tkjg}-1_{\{i=j\}})/ \left( 2Nu_i\mathcal{B}_{tik}(2Nu_j\mathcal{B}_{tjk}-1_{\{i=j\}})\right) \end{aligned}$$

and introduce the variables

161 $$\begin{aligned} \mathcal{V}_{tkij} = \left\{ \begin{array}{ll} E(\nu _{tki1}(\nu _{tki1}-1)|\varvec{\mathcal {B}}_t), &{} \text{ if } i=j,\\ E(\nu _{tki1}\nu _{tkj1}|\varvec{\mathcal {B}}_t), &{} \text{ if } i\ne j, \end{array}\right. \end{aligned}$$

which are conditional versions of $V_{kij}$ in (38). Then average with respect to the exchangeable random vectors $\{{\varvec{\nu }}_{tkg}\}_{g=1}^{2Nu_k}$ in (160) to deduce that

162 $$\begin{aligned} \mathcal{P}_{tijk}&= P(T=1|I_0=i,J_0=j,I_1=J_1 = k,T>0,\varvec{\mathcal {B}}_t)\nonumber \\&= 2Nu_k \mathcal{V}_{tkij}/\left( 2Nu_i\mathcal{B}_{tik}\cdot \left( 2N u_j\mathcal{B}_{tjk} -1_{\{i=j\}}\right) \right) \nonumber \\&= u_k \mathcal{V}_{tkij}/\left( 2Nu_i\mathcal{B}_{tik}u_j\mathcal{B}_{tjk}\left( 1-1_{\{i= j\}}(2Nu_i\mathcal{B}_{tik})^{-1}\right) \right) . \end{aligned}$$

We can rewrite (34) as

$$\begin{aligned} \mathcal{Q}_{tij,kl}=\mathcal{B}_{tik}\mathcal{B}_{tjl}\left( 1-\frac{1}{2Nu_i\mathcal{B}_{tik}} \right) ^{\{i=j,k=l\}}\left( \frac{1}{1-\frac{1}{2Nu_i}}\right) ^{\{i=j\}}, \end{aligned}$$

and taking the product of the last two displayed equations, we find that

163 $$\begin{aligned} \mathcal{P}_{tijk}\mathcal{Q}_{tij,kk}=\left( \frac{1}{1-\frac{1}{2Nu_i}}\right) ^{\{i=j\}} \frac{\mathcal{V}_{tkij}u_k}{2Nu_iu_j}, \end{aligned}$$

Formula (39) then follows from (157) by averaging with respect to $\varvec{\mathcal {B}}_t$ in (163), using that $E(\mathcal{V}_{tkij})=V_{kij}$, and finally dividing by $Q_{ij,kk}$. $\square$

### *Proof of Corollary 3*

Define ${\varvec{\phi }}_3$ as in (42), and the gene diversity vector ${\varvec{\mathcal{H}}}({\varvec{x}}) = \text{ vec }\left( (x_i(1-x_j)+x_j(1-x_i))_{i,j=1}^s\right) ^\prime$ of length $s^2$ obtained from an allele frequency vector ${\varvec{x}}$ of length $s$. Then

$$\begin{aligned} (P\phi _3)({\varvec{x}})&= \sum _{{\varvec{y}}} P({\varvec{x}},{\varvec{y}})\phi _3({\varvec{y}})\\&= \sum _{{\varvec{y}}} P({\varvec{x}},{\varvec{y}})\sum _{ij} \rho _{ij}\left( y_i(1-y_j) +y_j(1-y_i)\right) \\&= \sum _{ij} \rho _{ij}\sum _{{\varvec{y}}} P({\varvec{x}},{\varvec{y}})\left( y_i(1-y_j) +y_j(1-y_i)\right) \\&= \sum _{ij} \rho _{ij} E(\mathcal{H}_{tij}|{\varvec{X}}_{t-1}={\varvec{x}})\\&= \sum _{ij} \rho _{ij}\left( {\varvec{A}}{\varvec{\mathcal{H}}}({\varvec{x}})\right) _{ij}\\&= {\varvec{\rho }}{\varvec{A}}{\varvec{\mathcal{H}}}({\varvec{x}})\\&= \lambda {\varvec{\rho }}{\varvec{\mathcal{H}}}({\varvec{x}})\\&= \lambda \sum _{ij} \rho _{ij}\left( x_i(1-x_j)+x_j(1-x_i)\right) \\&= \lambda \phi _3({\varvec{x}}). \end{aligned}$$

In the fifth step we used (40), and in the seventh step that ${\varvec{\rho }}$ is a left eigenvector of ${\varvec{A}}$ with eigenvalue $\lambda$. Formula (43) is proved in the same way as the corresponding result for the right eigenvector ${\varvec{r}}$ of ${\varvec{A}}$ in Theorem 2. $\square$

**Verifying (** 63 **), (** 64 **) and (** 65 **)** We first insert (7) and the expression for $Q_{ij,kl}$ in (51) into (39), and find that $p_{ijk} = \left[ V_{kij}/(M_{ki}M_{kj})\right] / (2Nu_k-1_{\{i=j\}}u_k/u_i)$. Since $E(\nu _{tki1})=M_{ki}$, it follows from (38) that

164 $$\begin{aligned} p_{ijk} = \left\{ \begin{array}{ll} \left[ 1+ (\text{ Var }(\nu _{tki1})-M_{ki})/M_{ki}^2\right] /(2Nu_k-u_k/u_i), &{} i=j,\\ \left[ 1+ \text{ Cov }(\nu _{tki1},\nu _{tkj1})/(M_{ki}M_{kj})\right] /(2Nu_k), &{} i\ne j. \end{array}\right. \end{aligned}$$

In order to compute $\text{ Var }(\nu _{tki1})$ and $\text{ Cov }(\nu _{tki1},\nu _{tkj1})$ for $k=1$, the subpopulation of grandpaternally inherited alleles of the fathers of time $t-1$, let $\zeta _{tmmg} = \nu _{t11g} + \nu _{t21g^\prime }$ and $\zeta _{tmfg} = \nu _{t13g} + \nu _{t23g^\prime }$ be the total number of children that are males and females respectively, of the male in time $t-1$ whose paternally and maternally inherited genes have been assigned number $g$ and $g^\prime =g^\prime (g)$ from $1,\ldots ,N_m=2Nu_1$. (Due to the convention (9) that the first $2Nu_kX_{t-1,k}$ genes of subpopulation $k$ have the specified allele, we cannot assume $g^\prime =g$.) Due to exchangeability, $\tau _{mm}^2 = \text{ Var }(\zeta _{tmm1})$, $\tau _{mf}^2 = \text{ Var }(\zeta _{tmf1})$ and $\tau _{mm,mf} = \text{ Cov }(\zeta _{tmm1},\zeta _{tmf1})$.

It follows from Mendel’s law of inheritance that either the paternally or maternally inherited gamete is passed on to the offspring, with equal probability 0.5, independently between matings. Hence $\nu _{t11g}|\zeta _{tmmg} \sim \text{ Bin }(\zeta _{tmmg},0.5)$ and $\nu _{t13g}|\zeta _{tmfg} \sim \text{ Bin }(\zeta _{tmfg},0.5)$, which implies

$$\begin{aligned} \text{ Var }(\nu _{t11g})&= \text{ Var }(E(\nu _{t11g}|\zeta _{tmmg})) + E(\text{ Var }(\nu _{t11g}|\zeta _{tmmg}))\\&= \text{ Var }(\frac{1}{2}\zeta _{tmmg}) + E(\frac{1}{4}\zeta _{tmmg})\\&= \frac{1}{4}\text{ Var }(\zeta _{tmmg}) + \frac{1}{2}E(\nu _{t11g})\\&= \frac{1}{4}\tau _{mm}^2 + \frac{1}{2}M_{11}, \end{aligned}$$

and analogously $\text{ Var }(\nu _{t13g}) = \tau _{mf}^2/4 + M_{13}/2$ and $\text{ Cov }(\nu _{t11g},\nu _{t13g}) = \tau _{mm,mf}/4$. Inserting the last three formulas and (62) into (164), we see that $p_{111}$, $p_{331}$ and $p_{131}$ simplify to (63), (64) and (65) respectively. $\square$

### *Proof of Theorem 5*

Formulas (78) and (79) follow easily from a first order Taylor expansion of (73) with respect to $\varepsilon$ at 0, making use of (75) and (77). Next we apply Theorem 4. We want to show that (80) follows from (69), with $\varepsilon$ as in (74) and that (81) is obtained by inserting (79) into (72). Indeed,

$$\begin{aligned} C&= \sum _{ijk} \rho _{ij}(0)\frac{\sigma _{ijk}Q_{ij,kk}(0)}{u_k^\beta } - \sum _{ijkl}\rho _{ij}(0)\dot{Q}_{ij,kl}\\&+ 1_{\{\beta =1\}}\left( \sum _{ikl} \rho _{ii}(0) \frac{Q_{ii,kl} (0)}{u_i}- \sum _{ijk} \rho _{ij}(0) \frac{Q_{ij,kk}(0)}{u_k}\right) \\&= \sum _{ijk} \rho _{ij}(0) \frac{\sigma _{ijk}Q_{ij,kk}(0)}{u_k^\beta }, \end{aligned}$$

where in the last step we employed that ${\varvec{\rho }}(0)$ is a left eigenvector of ${\varvec{Q}}(0)$ with eigenvalue 1, and moreover that $\sum _{kl} Q_{ij,kl}(\varepsilon )=1$ for all $\varepsilon \ge 0$, which implies $\sum _{kl} Q_{ij,kl}(0)=1$ and $\sum _{kl} \dot{Q}_{ij,kl}=0$. $\square$

### *Proof of Corollary 5 and 6*

Corollary 4 implies that $Q_{ij,kl}(0)=B_{ik}B_{jl}$ in Corollary 6, so that

$$\begin{aligned} \sum _{ij} \gamma _i\gamma _j Q_{ij,kl}(0) = \sum _{ij} \gamma _i\gamma _j B_{ik}B_{jl} = \sum _i \gamma _i B_{ik}\sum _j \gamma _j B_{il} = \gamma _k\gamma _l \end{aligned}$$

follows from (4), and ${\varvec{\gamma }}\otimes {\varvec{\gamma }}= (\gamma _i\gamma _j)$ is a left eigenvector of ${\varvec{Q}}(0)$ with eigenvalue 1. This proves (89), and insertion into (81) yields (90). We then use (7) and (76) in order to prove (91), since

$$\begin{aligned} \sigma _{ijk} = \lim _{N\rightarrow \infty } \frac{V_{kij} u_k^2}{(Nu_k)^{1-\beta }u_i u_j (M_{ki}u_k/u_i)(M_{kj}u_k/u_j)}. \end{aligned}$$

In Corollary 5, it follows from Corollary 4 that $Q_{ii,kl}(0) = 1_{\{k=l\}}B_{ik}$, and

$$\begin{aligned} \sum _{ij} \gamma _i1_{\{i=j\}} Q_{ij,kl}(0) = \sum _i \gamma _i 1_{\{k=l\}}B_{ik} = 1_{\{k=l\}}\gamma _k, \end{aligned}$$

because of (4). This proves (86), which inserted into (81) yields (87). Since

$$\begin{aligned} V_{kii}&= E\left( \nu _{tki1}(\nu _{tki1}-1)\right) \\&= P(K_{ti}=k) E\left( \nu _{tki1}(\nu _{tki1}-1)|K_{ti}=k\right) \\&= B_{ik} \bar{V}_{kii} \end{aligned}$$

and $Q_{ii,kk}(0)=B_{ik}$, we get $\sigma _{iik} = \lim _{N\rightarrow \infty } \left( B_{ik}\bar{V}_{kii}u_k^2\right) /\left( (Nu_k)^{1-\beta }u_i^2B_{ik}\right)$ from (76), thereby proving (88). $\square$

**Motivation of Lemma** 1. We first sketch a proof of (110), using similar calculations as in Möhle (1998a). By the definition of an equilibrium distribution of a Markov chain, ${\varvec{B}}(\varepsilon )^t \rightarrow {\varvec{1}}{\varvec{\gamma }}(\varepsilon )$ as $t\rightarrow \infty$, where ${\varvec{1}}$ is a column vector of ones of length $s$. Hence we will study ${\varvec{B}}(\varepsilon )^t$ for large $t$ and use the approximation

165 $$\begin{aligned} {\varvec{B}}(\varepsilon )^t = \left( {\varvec{B}}(0)+\varepsilon \dot{{\varvec{B}}}\right) ^t \approx \sum _{r=0}^t {t\atopwithdelims ()r} \varepsilon ^r \left( {\varvec{B}}(0)^\infty \dot{{\varvec{B}}}{\varvec{B}}(0)^\infty \right) ^r, \end{aligned}$$

which can be shown to be accurate in the limit $\varepsilon \rightarrow 0$, with

$$\begin{aligned} {\varvec{B}}(0)^\infty = \left( \begin{array}{c@{\quad }c@{\quad }c} {\varvec{B}}_{11}(0)^\infty &{} \ldots &{} 0 \\ &{} \ddots &{} \\ 0 &{} \ldots &{} {\varvec{B}}_{mm}(0)^\infty \end{array}\right) = \left( \begin{array}{c@{\quad }c@{\quad }c} {\varvec{1}}_1{\varvec{\gamma }}_1 &{} \ldots &{} 0 \\ &{} \ddots &{} \\ 0 &{} \ldots &{} {\varvec{1}}_m{\varvec{\gamma }}_m \end{array}\right) \end{aligned}$$

a block diagonal matrix and ${\varvec{1}}_d=(1,\ldots ,1)^\prime$ a column vector of $|\mathcal{I}(d)|$ ones. It follows from (108) and ${\varvec{\gamma }}_d={\varvec{\gamma }}_d{\varvec{B}}_{dd}(0)$, that

$$\begin{aligned} \left( {\varvec{B}}(0)^\infty \dot{{\varvec{B}}}{\varvec{B}}(0)^\infty \right) ^r = \left( \begin{array}{c@{\quad }c@{\quad }c} G_{11}^{(r)}{\varvec{1}}_1{\varvec{\gamma }}_1 &{} \ldots &{} G_{1m}^{(r)}{\varvec{1}}_1{\varvec{\gamma }}_m \\ \vdots &{} &{} \vdots \\ G_{m1}^{(r)}{\varvec{1}}_m{\varvec{\gamma }}_1 &{} \ldots &{} G_{mm}^{(r)}{\varvec{1}}_m{\varvec{\gamma }}_m \end{array}\right) , \end{aligned}$$

where ${\varvec{G}}^r=(G_{ab}^{(r)})$. Putting $t=x/\varepsilon$ in (165), we notice that

$$\begin{aligned} {\varvec{B}}(\varepsilon )^{x/\varepsilon } \approx \left( \begin{array}{c@{\quad }c@{\quad }c} \exp (x{\varvec{G}})_{11} {\varvec{1}}_1{\varvec{\gamma }}_1 &{} \ldots &{} \exp (x{\varvec{G}})_{1m} {\varvec{1}}_1{\varvec{\gamma }}_m\\ \vdots &{} &{} \vdots \\ \exp (x{\varvec{G}})_{m1} {\varvec{1}}_m{\varvec{\gamma }}_1 &{} \ldots &{} \exp (x{\varvec{G}})_{mm} {\varvec{1}}_m{\varvec{\gamma }}_m, \end{array}\right) \end{aligned}$$

where

$$\begin{aligned} \exp (x{\varvec{G}}) = \sum _{r=0}^\infty \frac{x^r}{r!}{\varvec{G}}^r \mathop {\rightarrow }\limits ^{x\rightarrow \infty } \left( \begin{array}{c@{\quad }c@{\quad }c} \theta _1 &{} \ldots &{} \theta _m \\ \vdots &{} &{} \vdots \\ \theta _1 &{} \ldots &{} \theta _m \end{array}\right) , \end{aligned}$$

since ${\varvec{\theta }}=(\theta _1,\ldots ,\theta _m)$ is an equilibrium distribution of the continuous time Markov process with infinitesimal generator ${\varvec{G}}$. Combining the last two displayed equations, we find for large $x$ and small $\varepsilon$ that

$$\begin{aligned} {\varvec{B}}(\varepsilon )^{x/\varepsilon } \approx \left( \begin{array}{c@{\quad }c@{\quad }c} \theta _1 {\varvec{1}}_1{\varvec{\gamma }}_1 &{} \ldots &{} \theta _m{\varvec{1}}_1{\varvec{\gamma }}_m \\ \vdots &{} &{} \vdots \\ \theta _1 {\varvec{1}}_m{\varvec{\gamma }}_1 &{} \ldots &{} \theta _m{\varvec{1}}_m{\varvec{\gamma }}_m \end{array}\right) = {\varvec{1}}{\varvec{\gamma }}(0), \end{aligned}$$

where ${\varvec{\gamma }}(0)=(\gamma _i(0))$. We use (106), (108) and (110) to prove (111);

$$\begin{aligned} B(\varepsilon )&= \sum _{d=1}^m \sum _{i\in \mathcal{I}(d)}\gamma _i(\varepsilon )\sum _{k\notin \mathcal{I}(d)} B_{ik}(\varepsilon )\\&= \varepsilon \sum _{d=1}^m \sum _{i\in \mathcal{I}(d)}\gamma _i(\varepsilon )\sum _{k\notin \mathcal{I}(d)} \dot{B}_{ik}\\&= \varepsilon \sum _{d=1}^m \sum _{i\in \mathcal{I}(d)}\gamma _i(0)\sum _{k\notin \mathcal{I}(d)} \dot{B}_{ik} + o(\varepsilon )\\&= - \varepsilon \sum _{d=1}^m \sum _{i,k\in \mathcal{I}(d)}\gamma _i(0)\dot{B}_{ik} + o(\varepsilon ), \end{aligned}$$

since the row sums of $\dot{B}$ are zero. Finally, (112) follows from (105), since

$$\begin{aligned} M(\varepsilon )&= \sum _{d=1}^m\sum _{k\in \mathcal{I}(d)}u_k(\varepsilon )\sum _{i\notin \mathcal{I}(d)} M_{ki}(\varepsilon )\\&= \varepsilon \sum _{d=1}^m \sum _{k\in \mathcal{I}(d)} u_k(0)\sum _{i\notin \mathcal{I}(d)} \dot{M}_{ki} + o(\varepsilon ). \end{aligned}$$

$$\square $$

**Proving (** 133 **).** By (105) and (132), the off-diagonal block elements of $\dot{{\varvec{B}}}$ are

$$\begin{aligned} \begin{array}{llll} \dot{B}_{1,z+k} &{}=&{} c_1B_{z+1,z+k}(0)u_{z+1}, &{} k=1,\ldots ,z,\\ \dot{B}_{k+1,z+k} &{}=&{} c_{k+1}u_{z+k+1}, &{} k=1,\ldots ,z-1,\\ \dot{B}_{z+1,k} &{}=&{} c_1B_{1k}(0)u_1, &{} k=1,\ldots ,z,\\ \dot{B}_{z+k+1,k} &{}=&{} c_{k+1}u_{k+1}, &{} k=1,\ldots ,z-1. \end{array} \end{aligned}$$

where $B_{1k}(0)$ is the probability that the parent of a newborn in deme 1 originates from age class $k$ of that deme when there is no migration, and similarly $B_{z+1,z+k}(0)$ is the probability that the parent of a newborn in deme 2 has a parent from age class $k$ of that deme. Invoking (108), the elements of ${\varvec{G}}$ are

$$\begin{aligned} G_{12}&= - G_{11} = \sum _{i=1}^z \gamma _{1i}\sum _{k=1}^z \dot{B}_{i,z+k} = \sum _{i=1}^z \gamma _{1i}c_iu_{z+i},\\ G_{21}&= - G_{22} = \sum _{i=1}^z \gamma _{2i}\sum _{k=1}^z \dot{B}_{z+i,k} = \sum _{i=1}^z \gamma _{2i}c_iu_{i}. \end{aligned}$$

Use (119) to deduce that $\dot{{\varvec{\Lambda }}}=(\dot{\Lambda }_{\alpha ,\beta })_{\alpha ,\beta =12,21}$ is a diagonal matrix of order $v=2(2-1)=2$, with both diagonal elements equal to $G_{11}+G_{22}$. Therefore

166 $$\begin{aligned} \lambda _{ \text{ max }}(\dot{{\varvec{\Lambda }}}) = G_{11}+G_{22} = -\sum _{i=1}^z c_i(\gamma _{1i}u_{z+i}+\gamma _{2i}u_i). \end{aligned}$$

Because of (8) and (132), the numerator of (122) equals

167 $$\begin{aligned}&\sum _{k=1}^z u_k \sum _{i=1}^z \dot{M}_{k,z+i} + \sum _{k=1}^z u_{z+k} \sum _{i=1}^z \dot{M}_{z+k,i}\nonumber \\&\quad = \sum _{k=1}^z u_k\left( c_1M_{k1}u_{z+1} + c_{k+1}M_{k,k+1} u_{z+k+1}1_{\{k<z\}}\right) \nonumber \\&\qquad + \sum _{k=1}^z u_{z+k}\left( c_1M_{z+k,z+1}u_{1} + c_{k+1} M_{z+k,z+k+1}u_{k+1}1_{\{k<z\}}\right) \nonumber \\&\quad = 2 \sum _{k=1}^z c_ku_ku_{z+k}. \end{aligned}$$

Inserting (166) and (167) into (122), we arrive at (133). $\square$
